# Supplementary figures and images for: High resolution assessment of commercial fisheries activity along the US West Coast using Vessel Monitoring System data with a case study using California groundfish fisheries
Source: PLoS One. 2024 Jun 6;19(6):e0298868. doi: 10.1371/journal.pone.0298868 (PMC11156284; doi:10.1371/journal.pone.0298868)

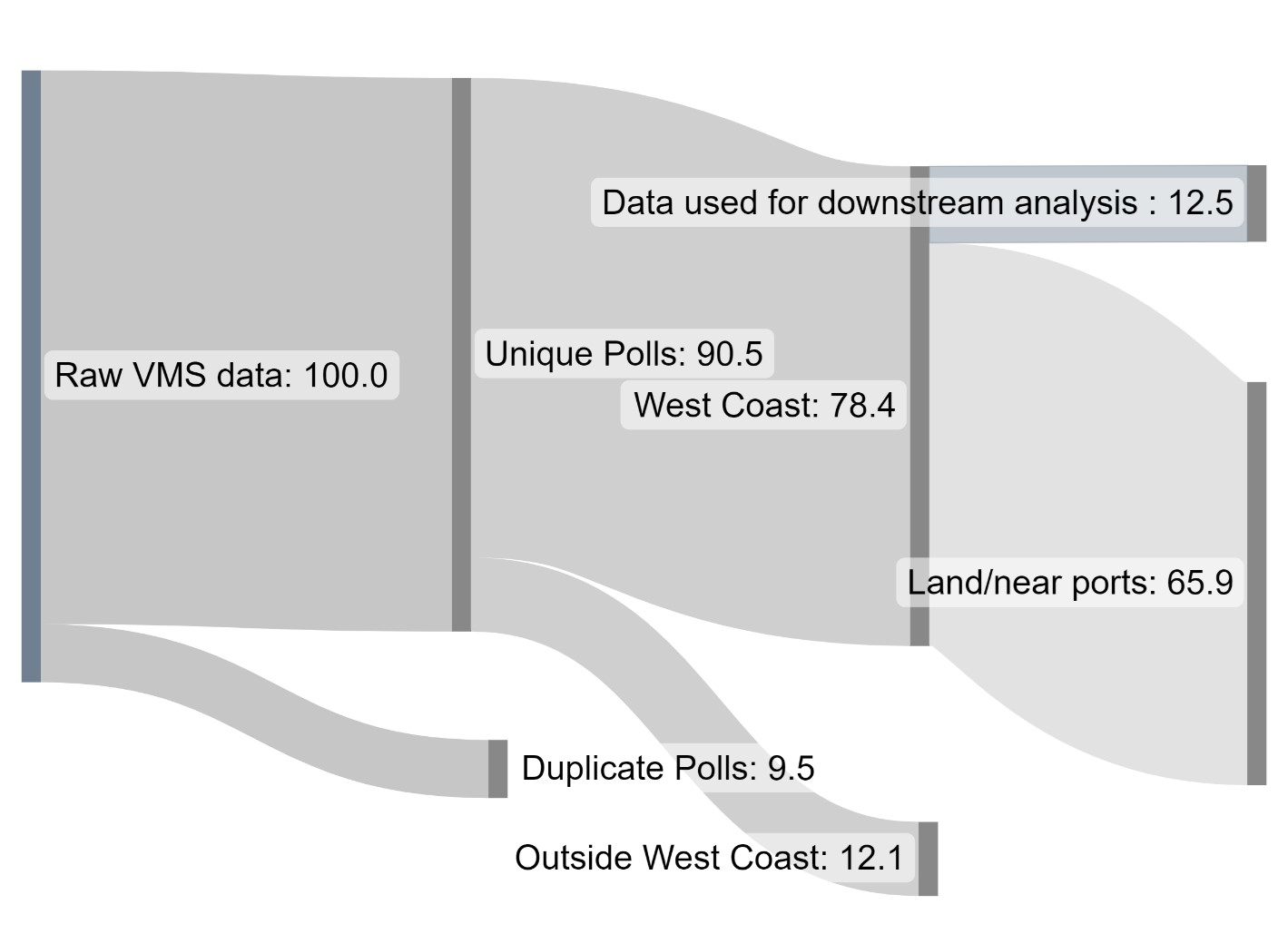

Supplement: S1 Fig — The starting point in the diagram is the raw data after correcting negative latitudes (0.39% of raw data). The percentage of data relative to the raw data for each step is provided. The raw data included over 50 million VMS polls, so the data for downstream analysis of fishing activity (12.5% of the VMS polls) still included over 6 million individual VMS polls. (TIF) [file pone.0298868.s001.tif]

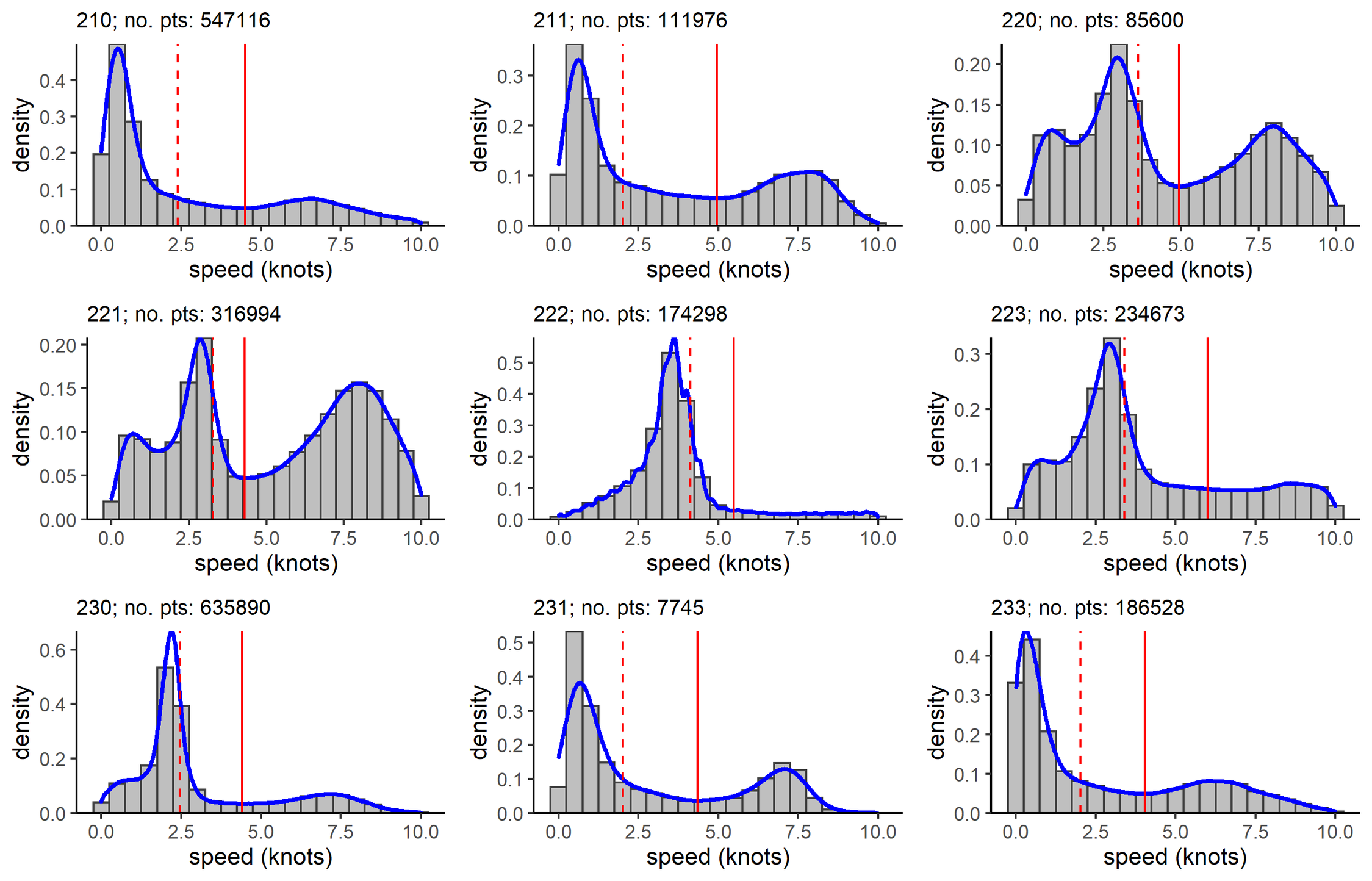

Supplement: S2 Fig — The blue line represents a kernel density fit of the data. The red solid line shows the local minimum of the distribution and the red dashed line shows the slope minimum between two and six knots. The number in the title for each subplot is the declaration code, and no. pts is the number of VMS polls included in each declaration code. (TIF) [file pone.0298868.s002.tif]

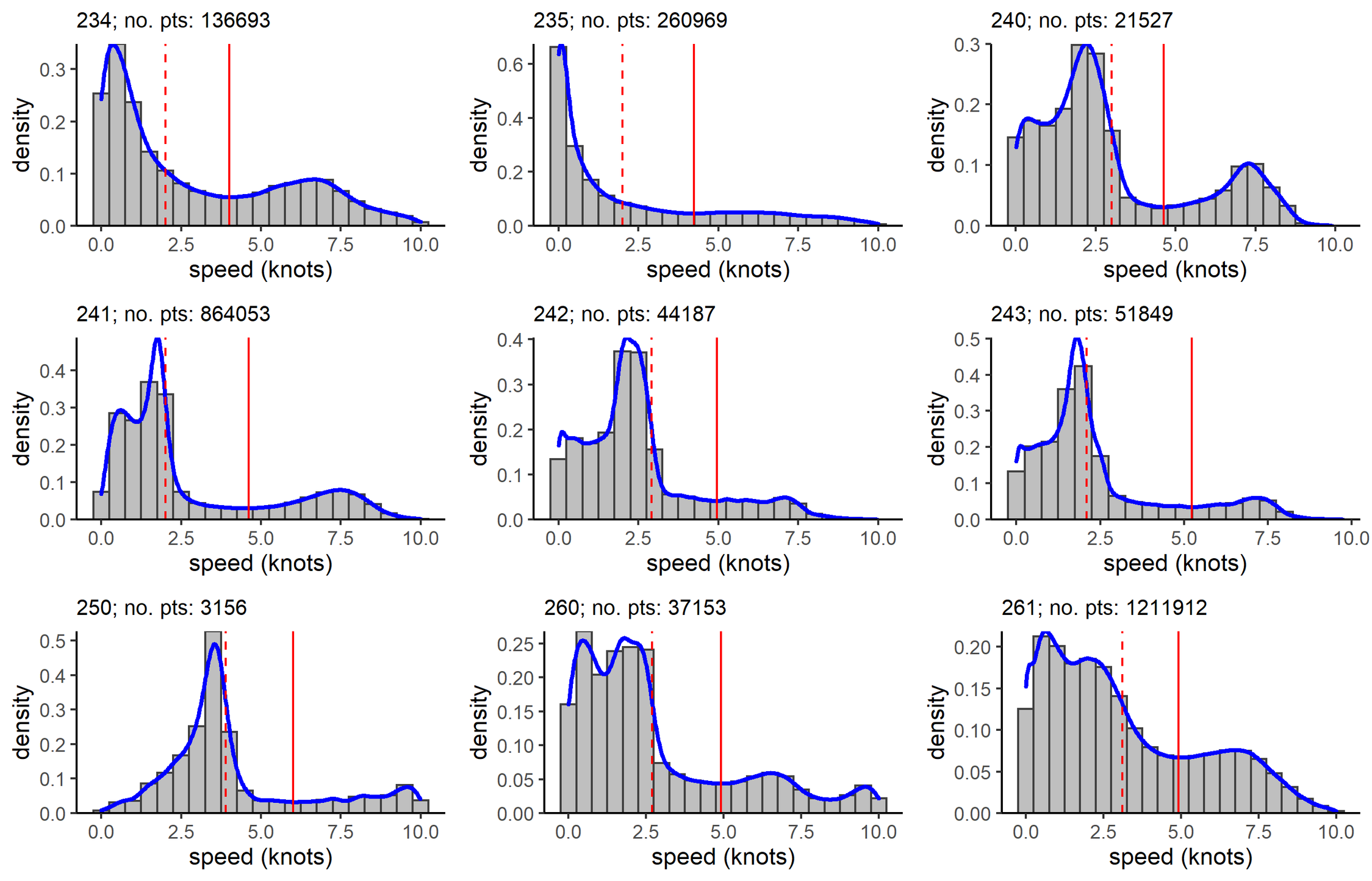

Supplement: S3 Fig — Similar to S2 Fig, but for Declaration Codes 234–261. (TIF) [file pone.0298868.s003.tif]

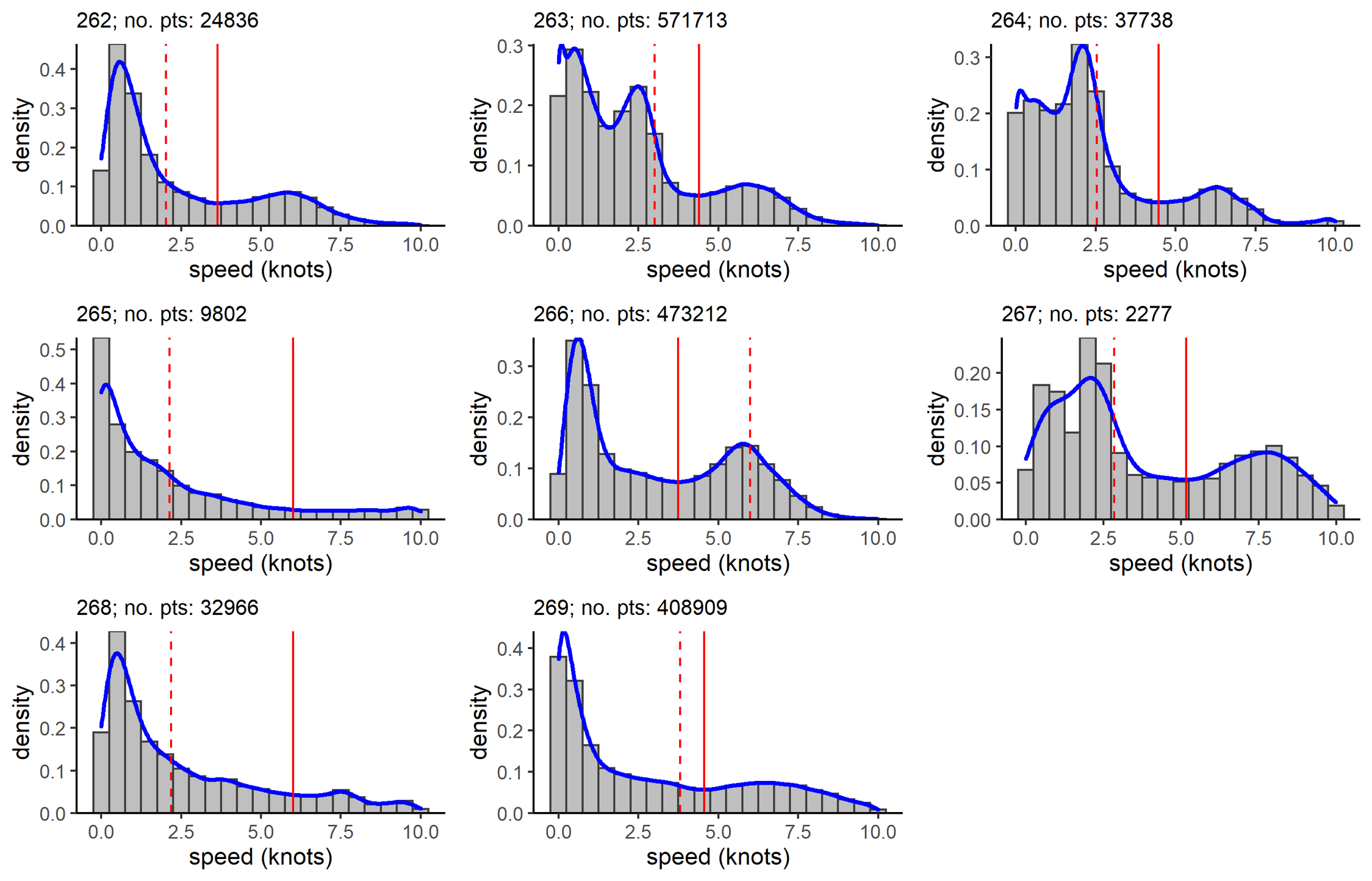

Supplement: S4 Fig — Similar to S2 Fig, but for Declaration Codes 262–269. (TIF) [file pone.0298868.s004.tif]

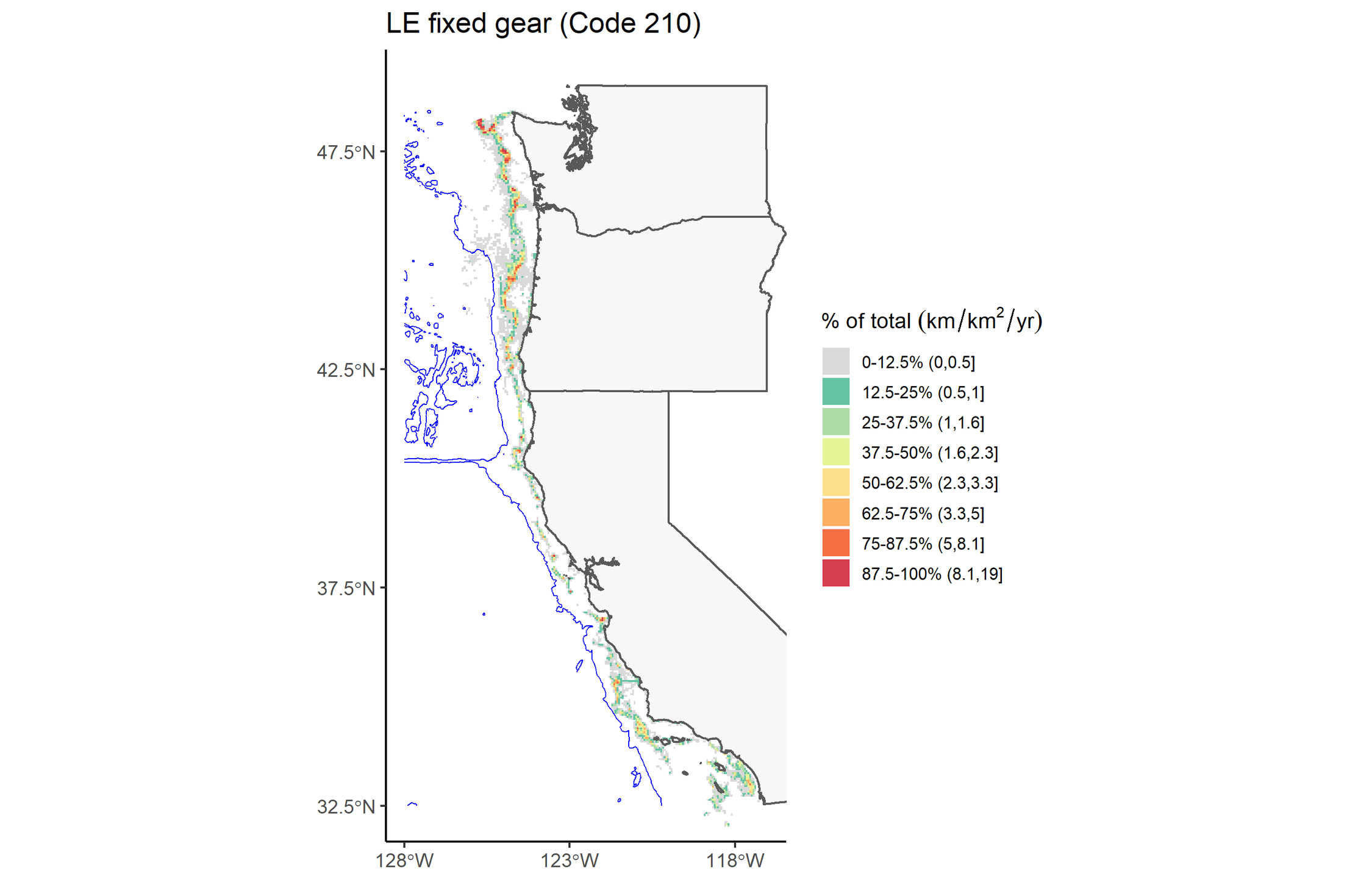

Supplement: S5 Fig — The blue line is the 2700 m isobath, representing the maximum depth for all groundfish fisheries except for midwater trawl. The area outside of the biological depth limit may represent erroneous declaration codes, and only represent 2.6% of the total effort for the fishery. (TIF) [file pone.0298868.s005.tif]

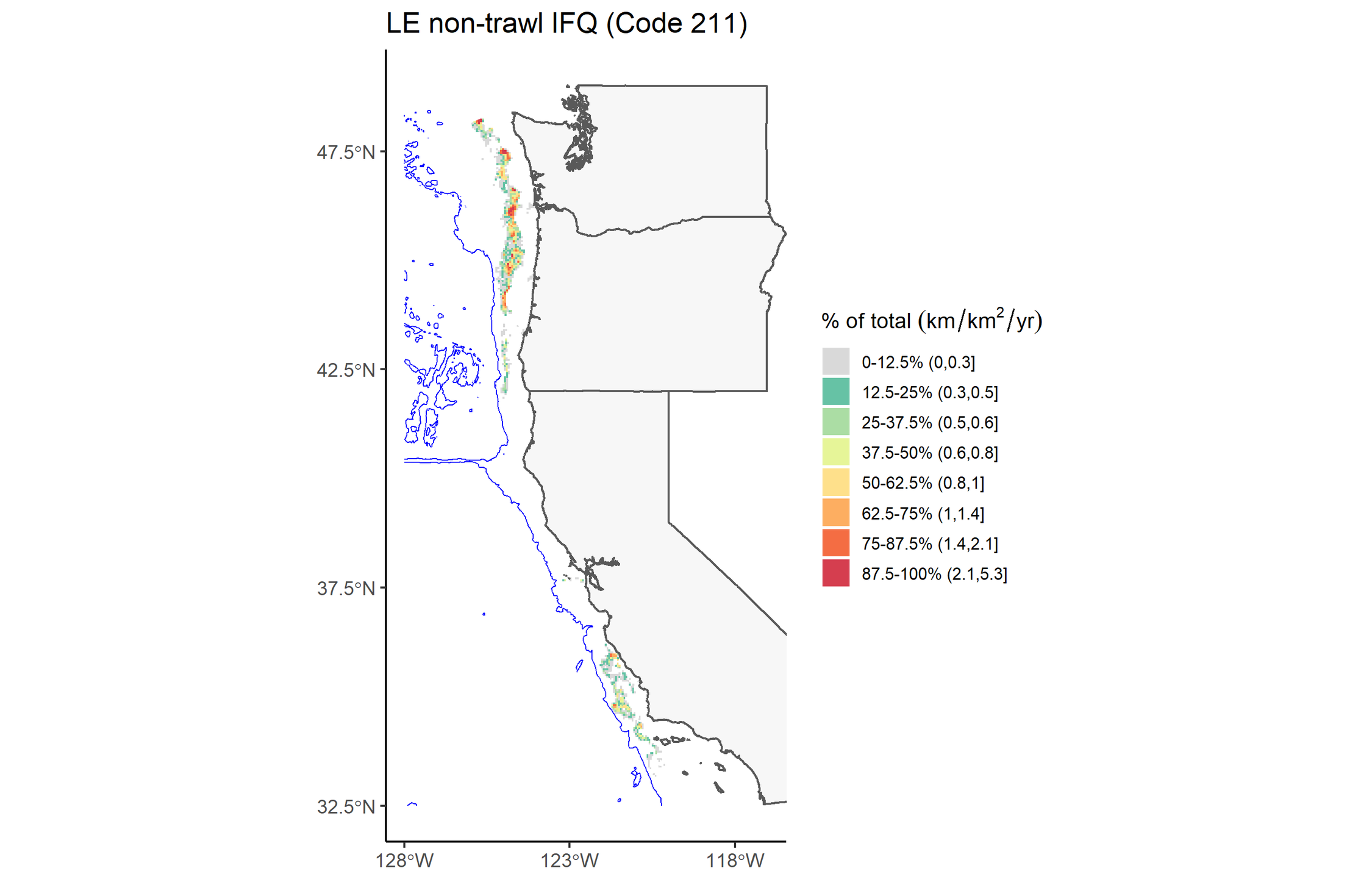

Supplement: S6 Fig — The area outside of the biological depth limit may represent erroneous declaration codes, and only represent 0.8% of the total effort for the fishery. (TIF) [file pone.0298868.s006.tif]

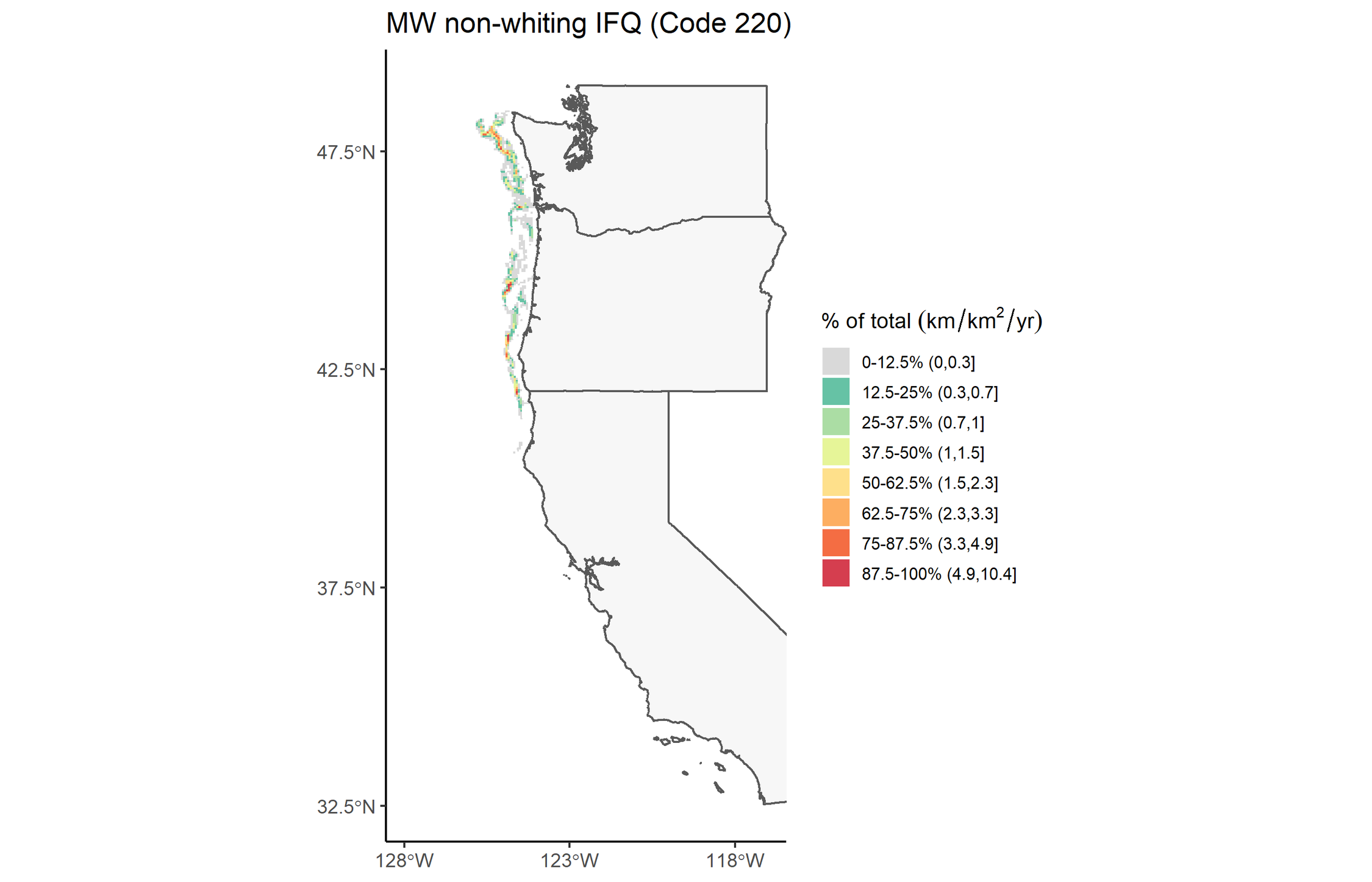

Supplement: S7 Fig — An isobath line is not added since it is not applicable to the midwater trawl fishery. (TIF) [file pone.0298868.s007.tif]

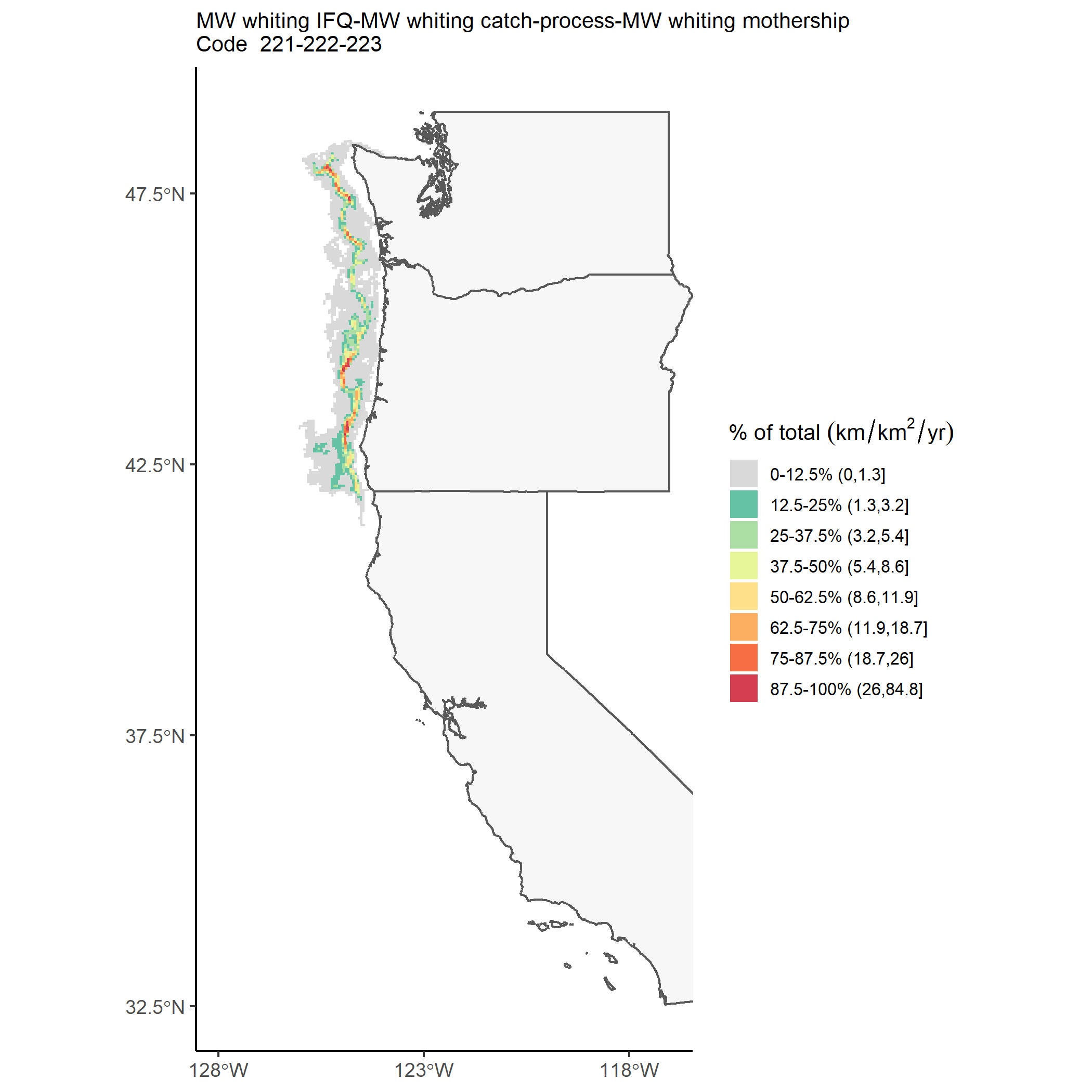

Supplement: S8 Fig — An isobath line is not including since depth limits are not applicable to the midwater trawl fisheries. (TIF) [file pone.0298868.s008.tif]

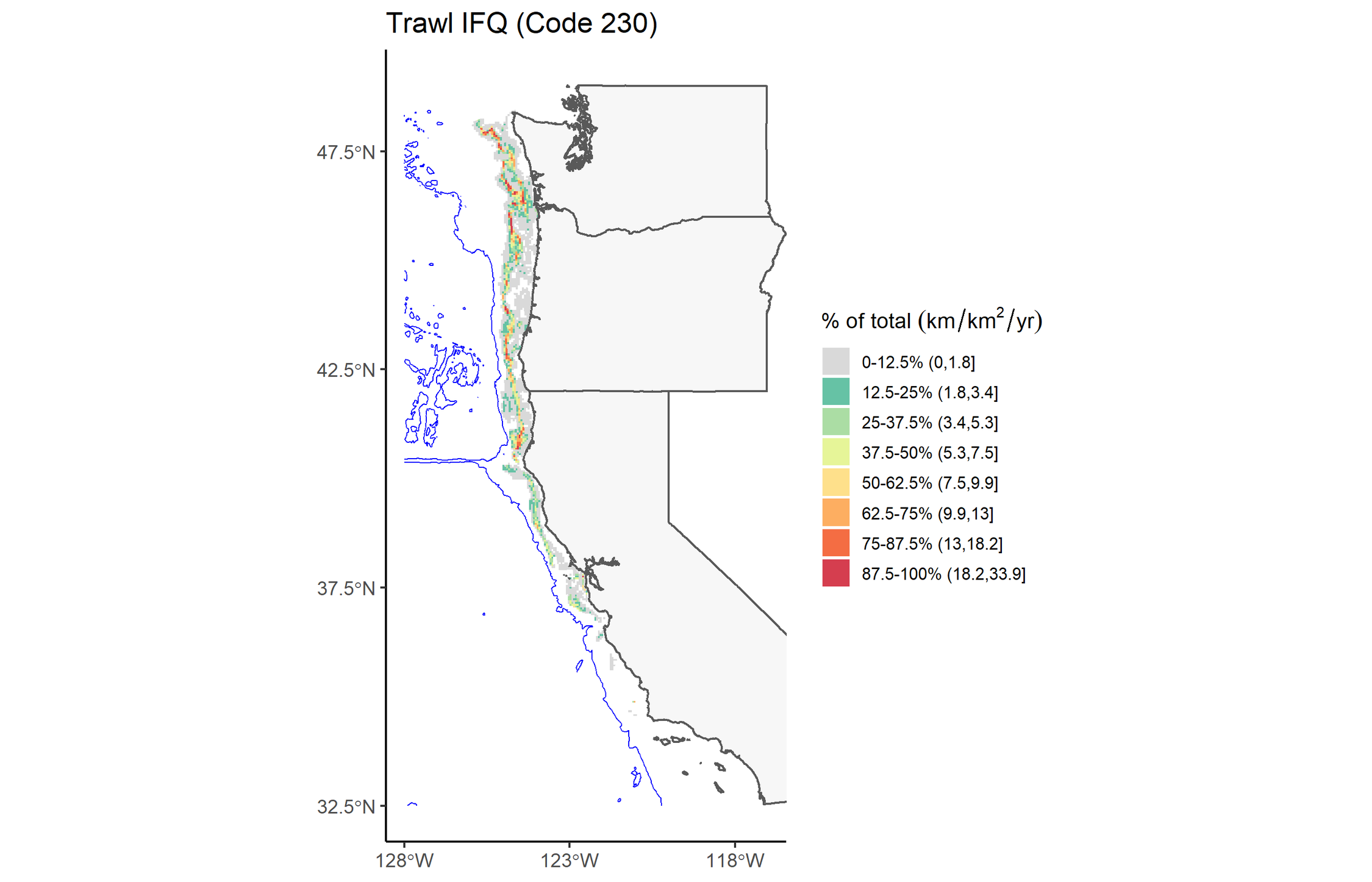

Supplement: S9 Fig — Almost all of the data (~100%) are inside the biological depth limit for the species. (TIF) [file pone.0298868.s009.tif]

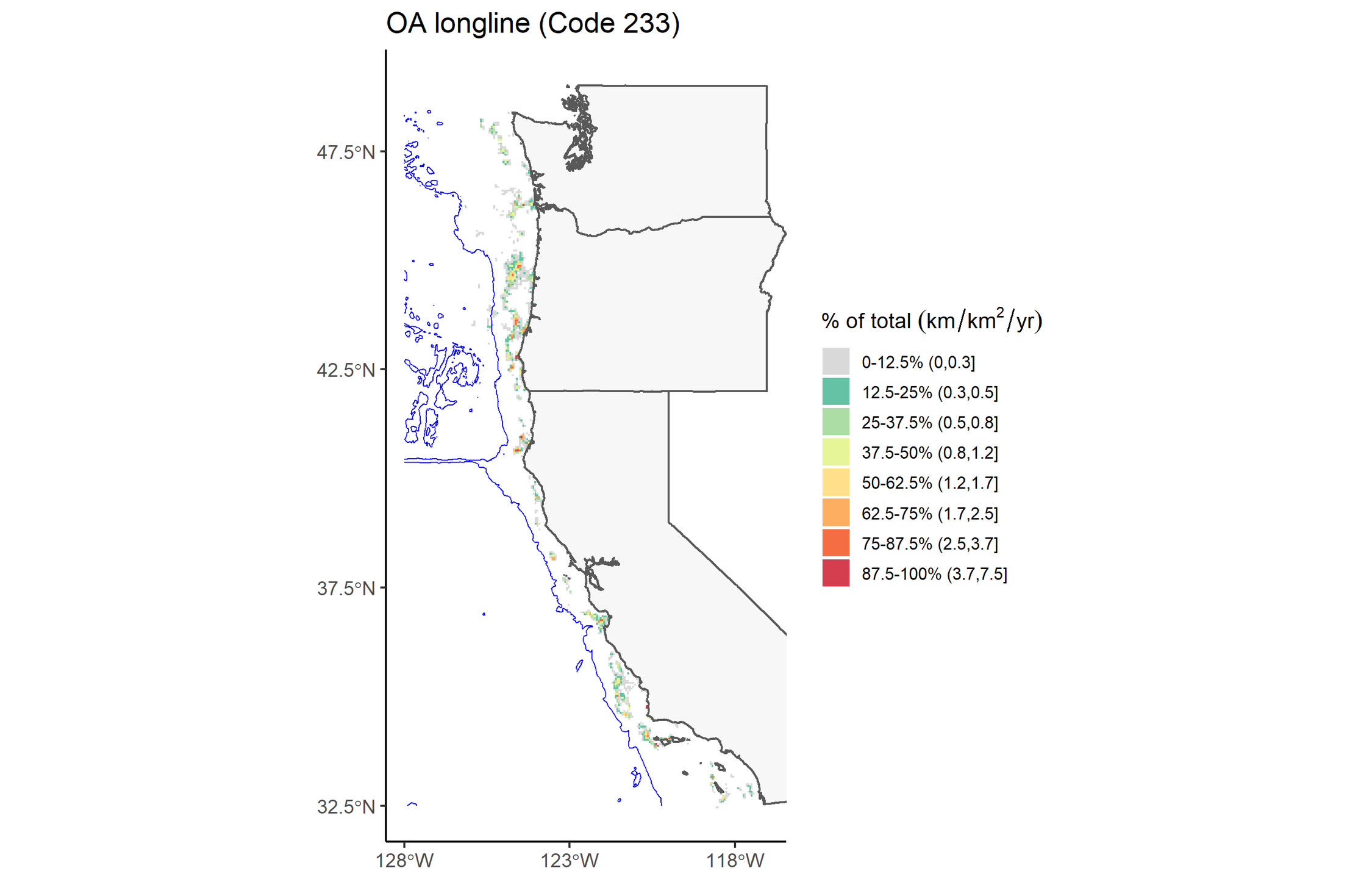

Supplement: S10 Fig — The area outside of the biological depth limit may represent erroneous declaration codes, and only represent 2.7% of the total effort for the fishery. (TIF) [file pone.0298868.s010.tif]

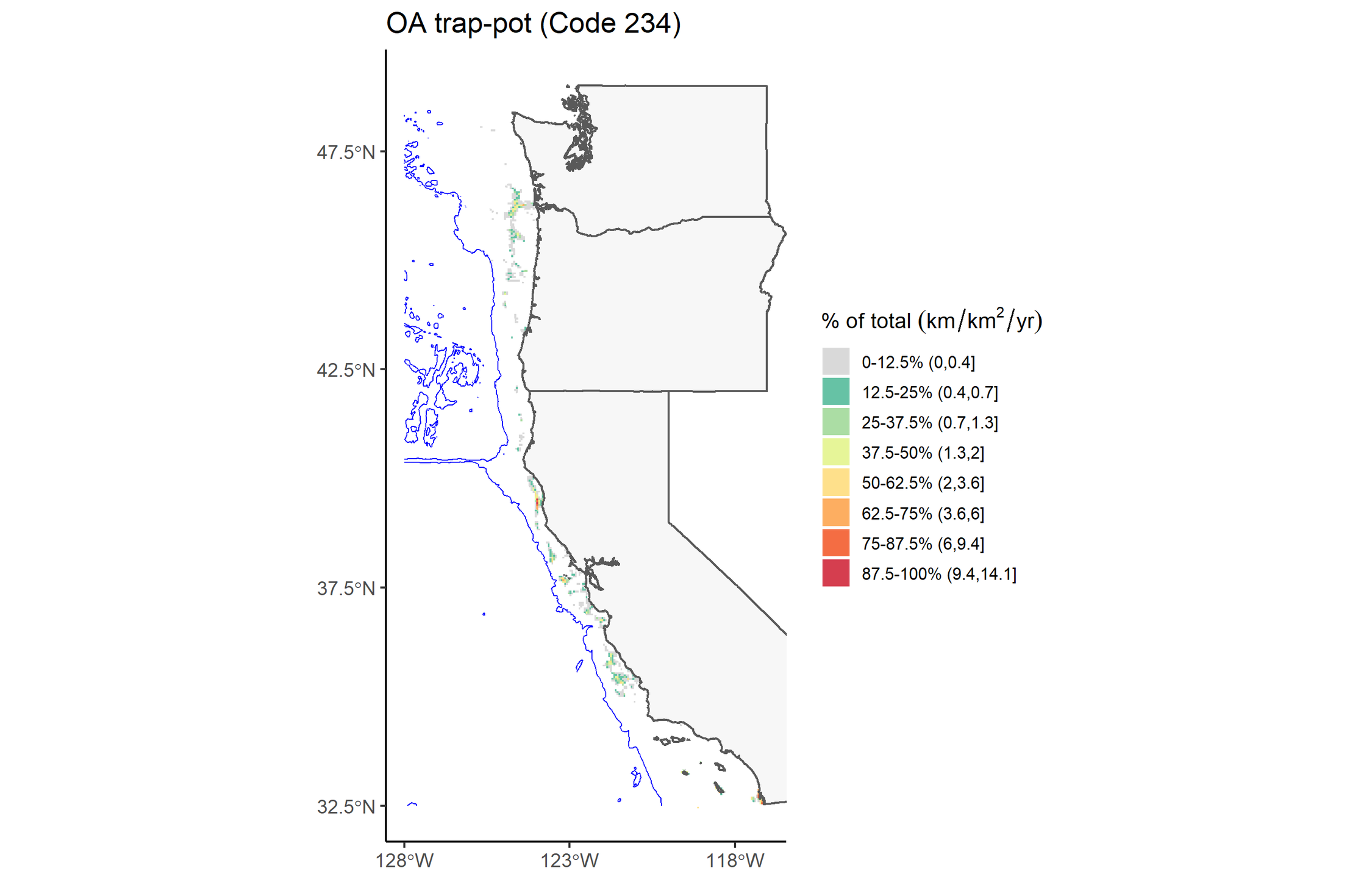

Supplement: S11 Fig — The area outside of the biological depth limit may represent erroneous declaration codes, and only represent 1.1% of the total effort for the fishery. (TIF) [file pone.0298868.s011.tif]

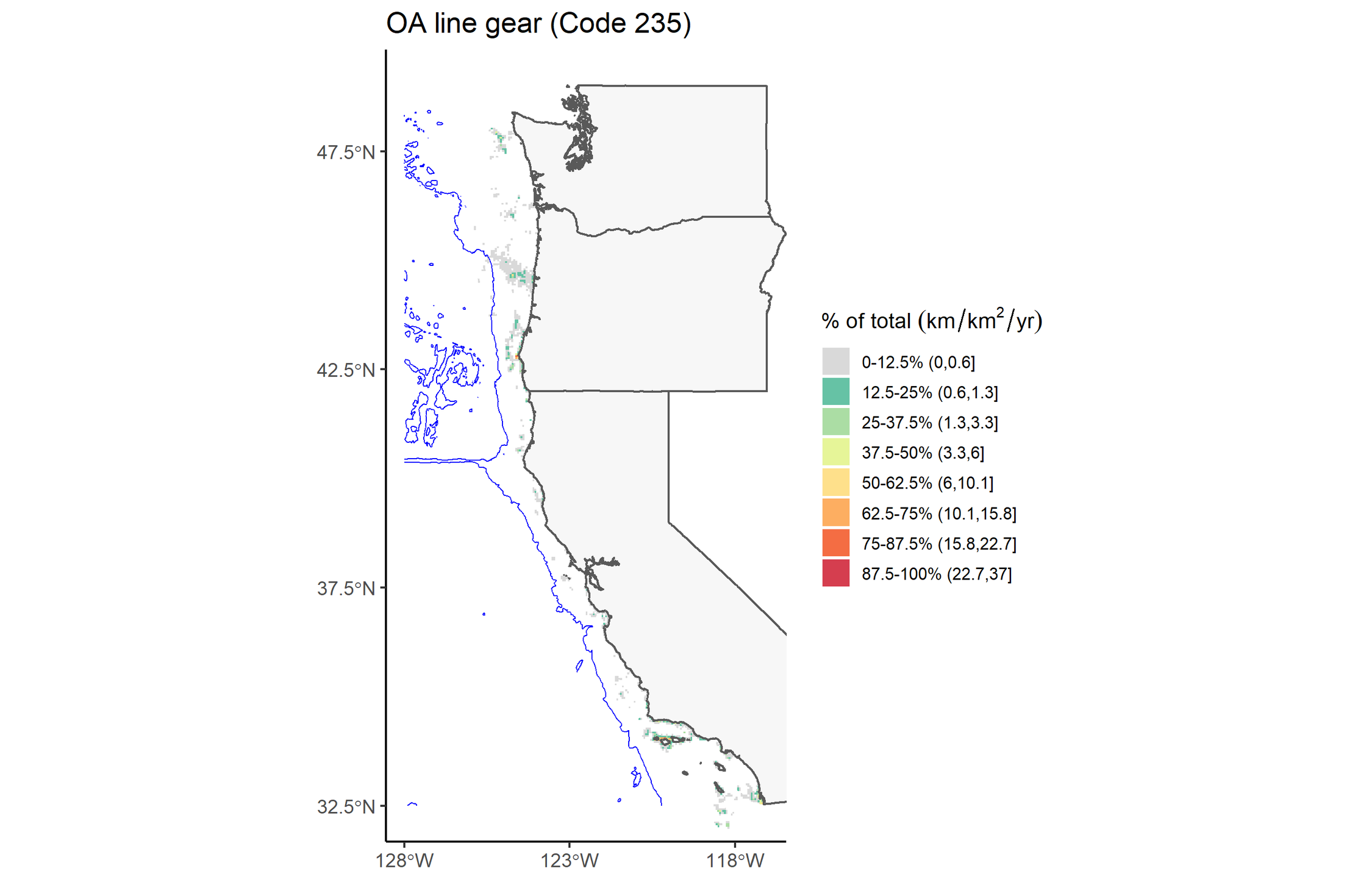

Supplement: S12 Fig — The area outside of the biological depth limit may represent erroneous declaration codes, and only represent 6% of the total effort for the fishery. (TIF) [file pone.0298868.s012.tif]

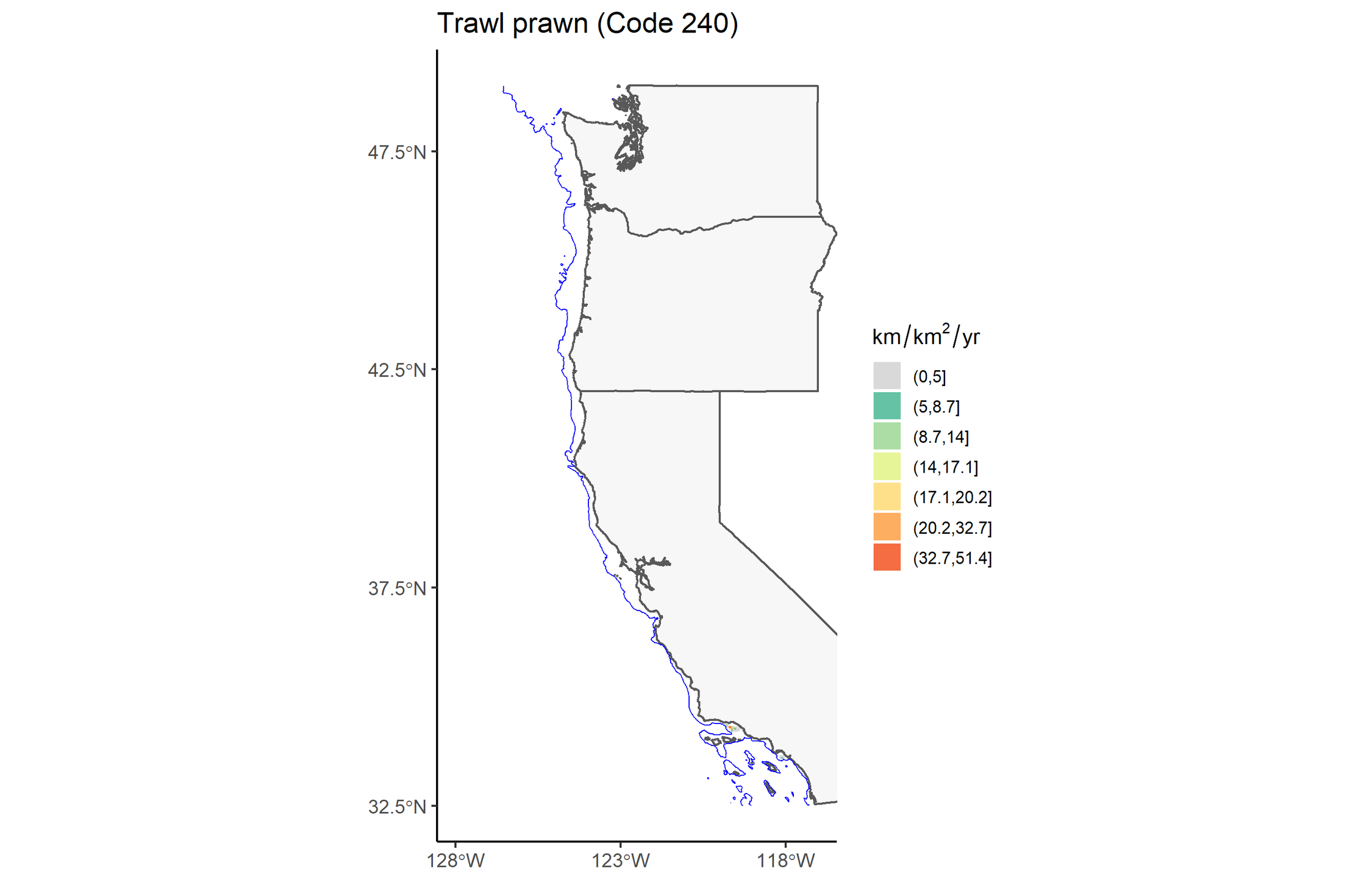

Supplement: S13 Fig — The blue line is the 300 m isobath, representing the maximum biological depth for the target species. The area outside of the biological depth limit may represent erroneous declaration codes, and only represent 0.4% of the total effort for the fishery. Data were too sparse to generate octiles as in the other plots. (TIF) [file pone.0298868.s013.tif]

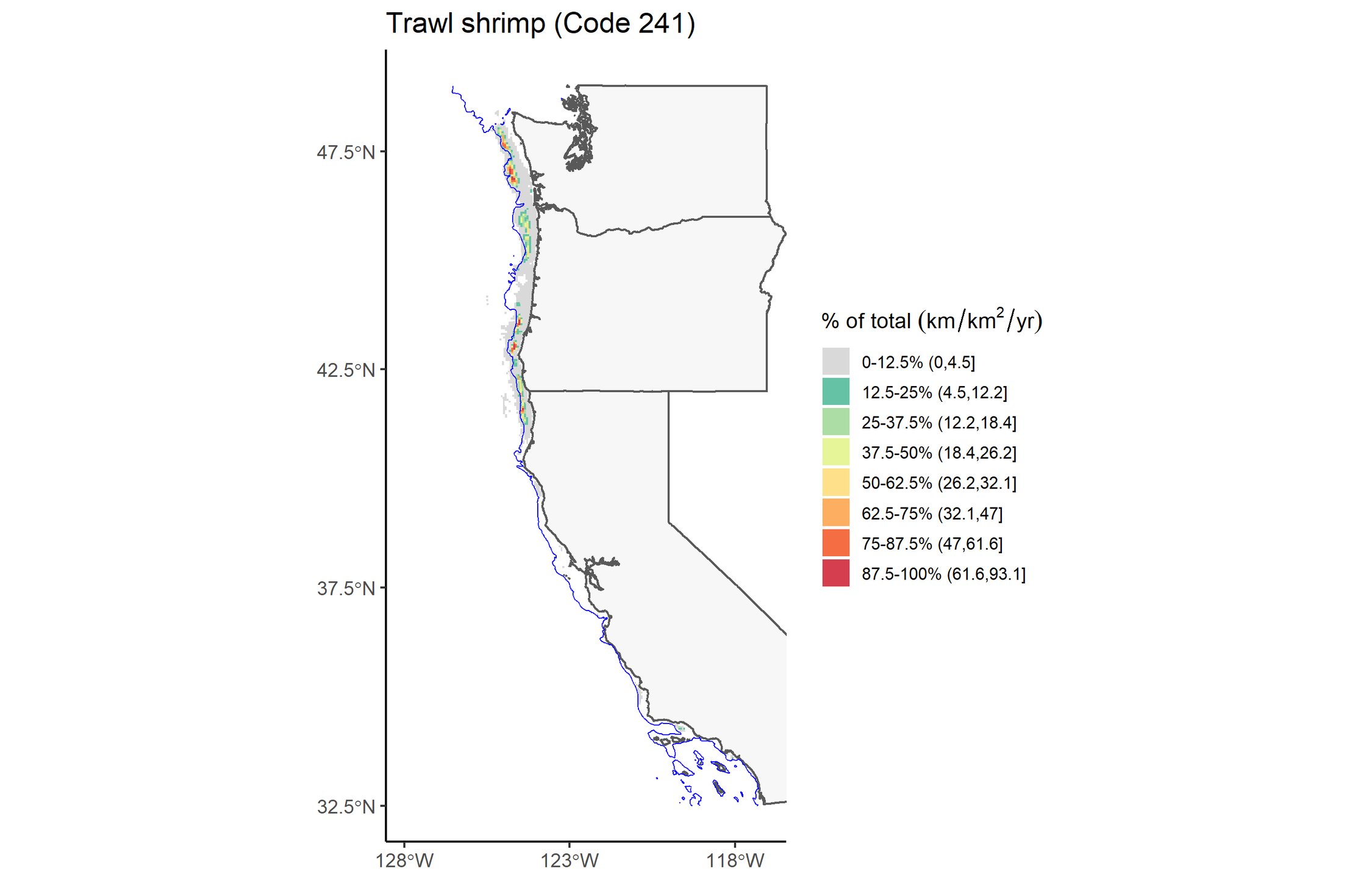

Supplement: S14 Fig — The blue line is the 300 m isobath, representing the maximum biological depth for the target species. The area outside of the biological depth limit may represent erroneous declaration codes, and only represent 1.7% of the total effort for the fishery. (TIF) [file pone.0298868.s014.tif]

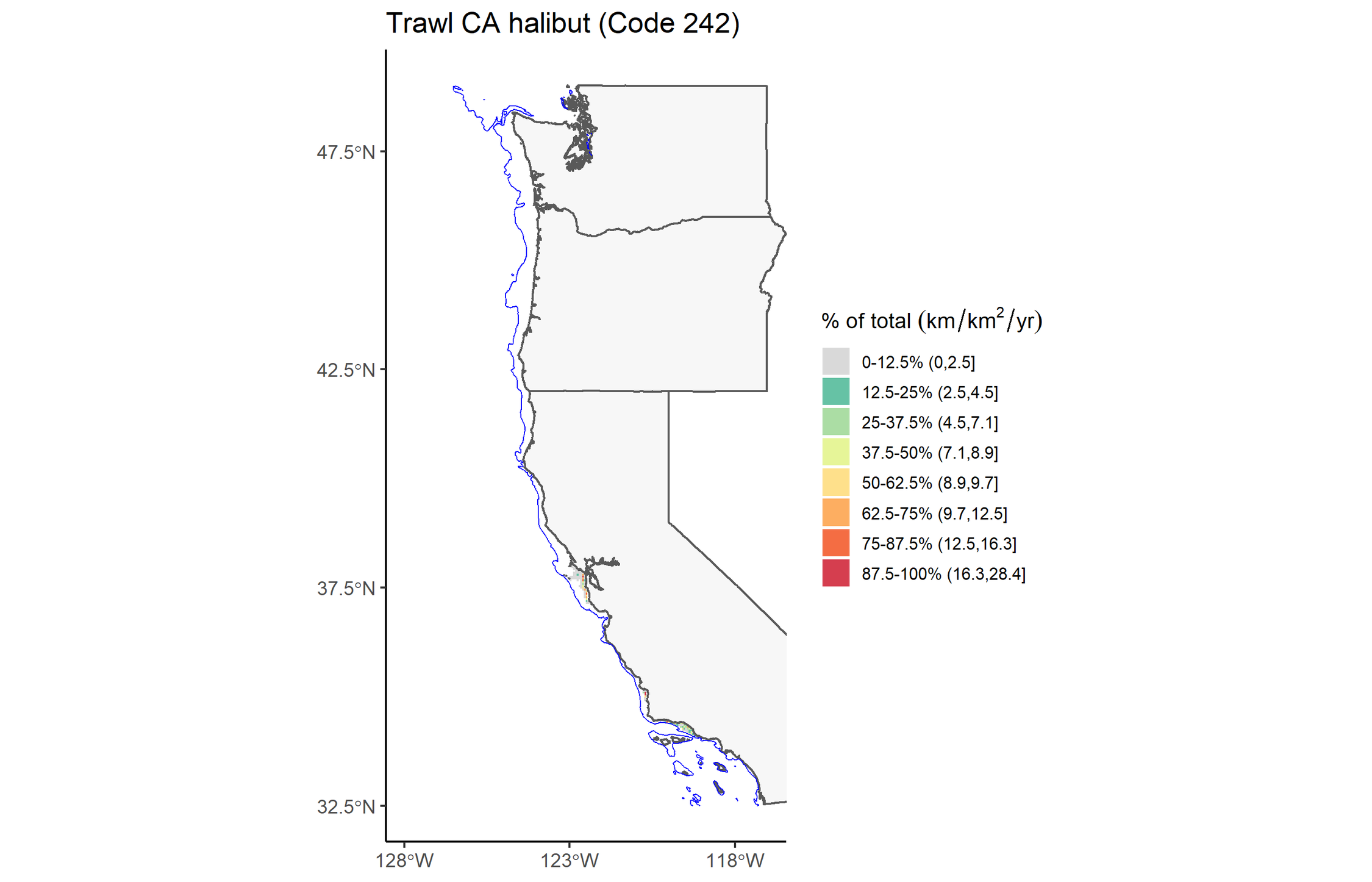

Supplement: S15 Fig — The blue line is the 200 m isobath, representing the maximum biological depth for the target species. The area outside of the biological depth limit may represent erroneous declaration codes, and only represent 0.4% of the total effort for the fishery. (TIF) [file pone.0298868.s015.tif]

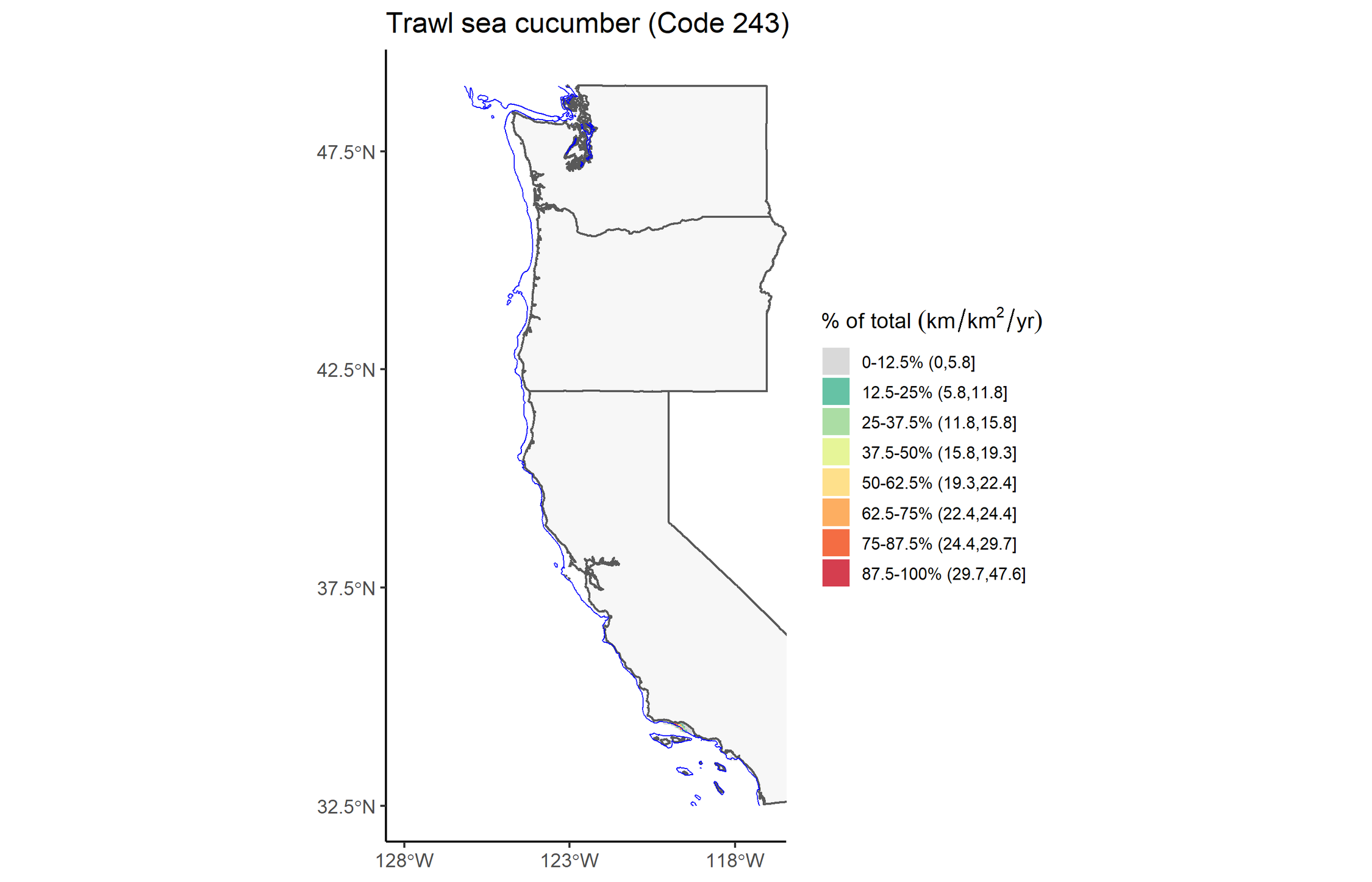

Supplement: S16 Fig — The blue line is the 100 m isobath, representing the maximum biological depth for the target species. The area outside of the biological depth limit may represent erroneous declaration codes, and represent 10.3% of the total effort for the fishery. (TIF) [file pone.0298868.s016.tif]

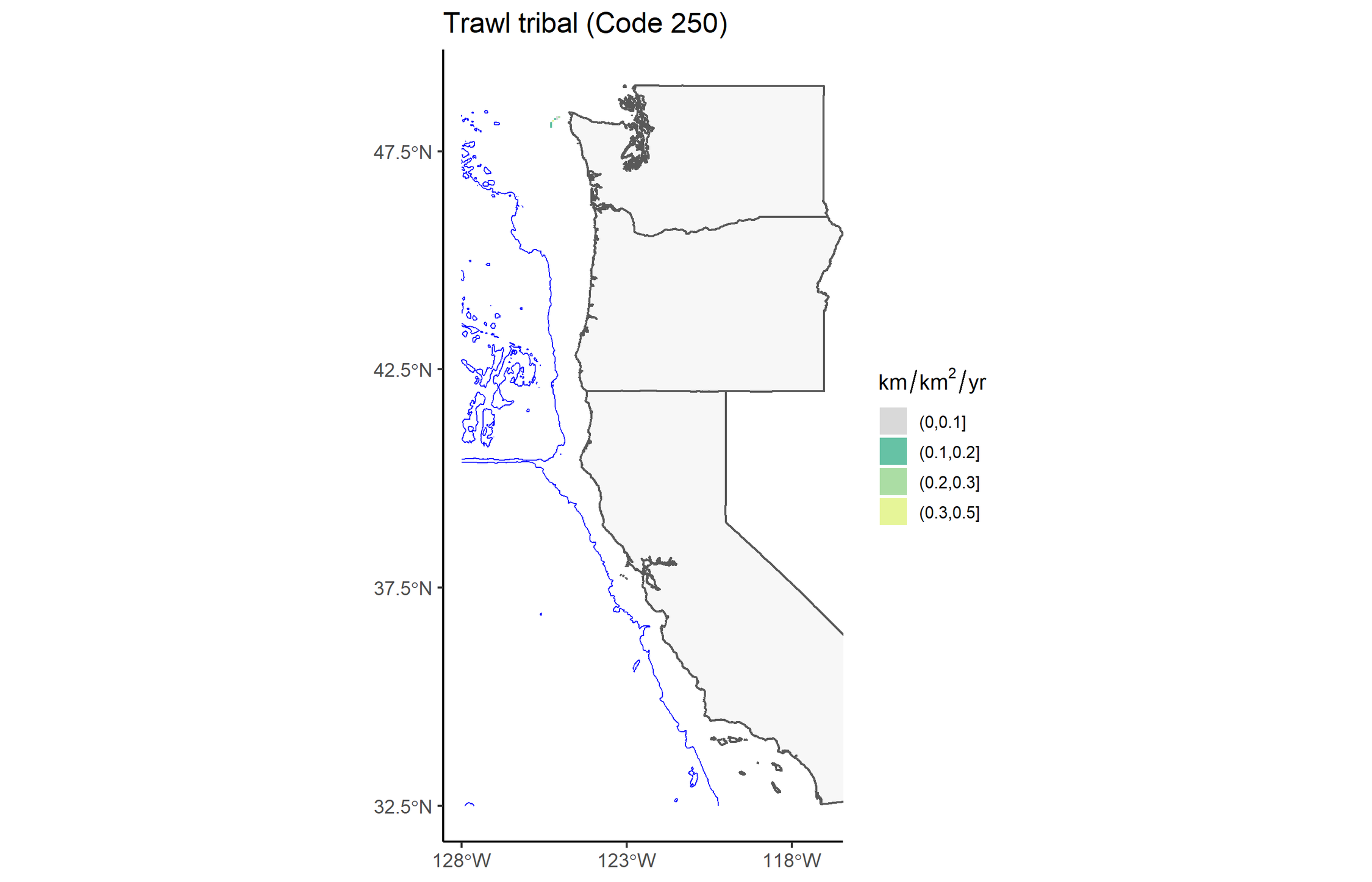

Supplement: S17 Fig — All of the data (100%) are inside the biological depth limit for the species. Data were too sparse to generate octiles as in the other plots. (TIF) [file pone.0298868.s017.tif]

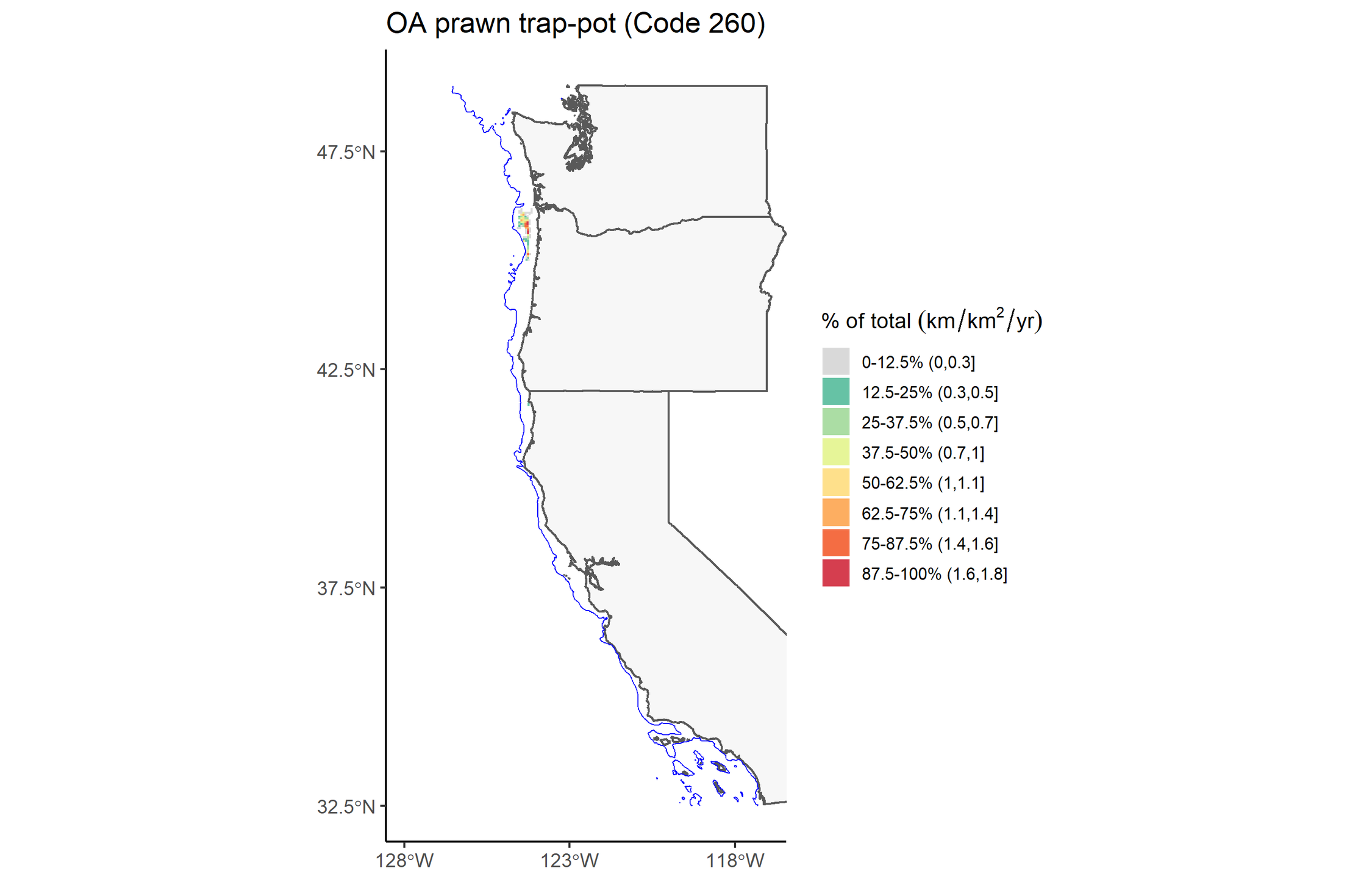

Supplement: S18 Fig — The blue line is the 300 m isobath, representing the maximum biological depth for the target species. The area outside of the biological depth limit may represent erroneous declaration codes, and only represent 3.2% of the total effort for the fishery. (TIF) [file pone.0298868.s018.tif]

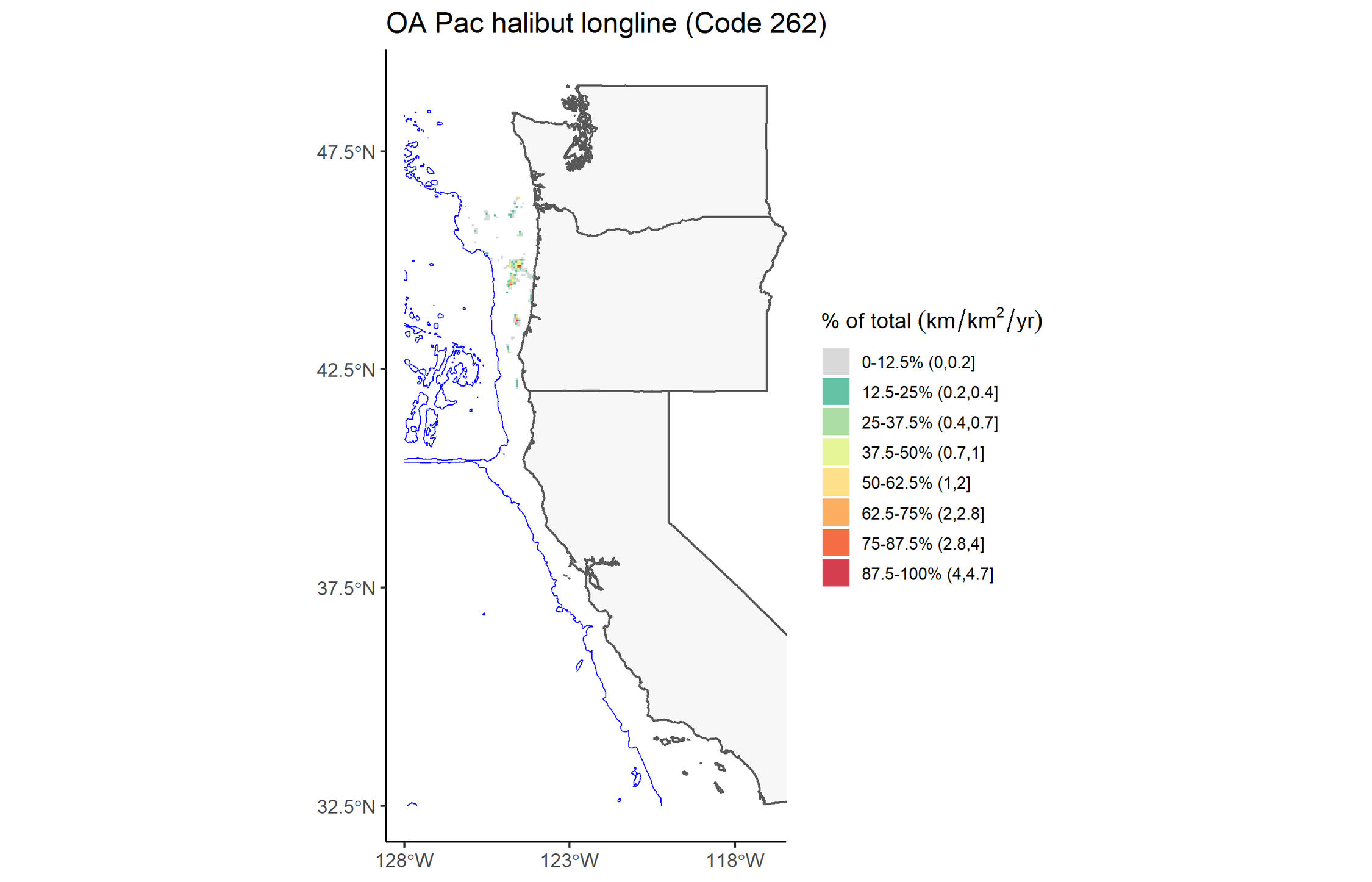

Supplement: S19 Fig — The area outside of the biological depth limit may represent erroneous declaration codes and represent 4.9% of the total effort for the fishery. (TIF) [file pone.0298868.s019.tif]

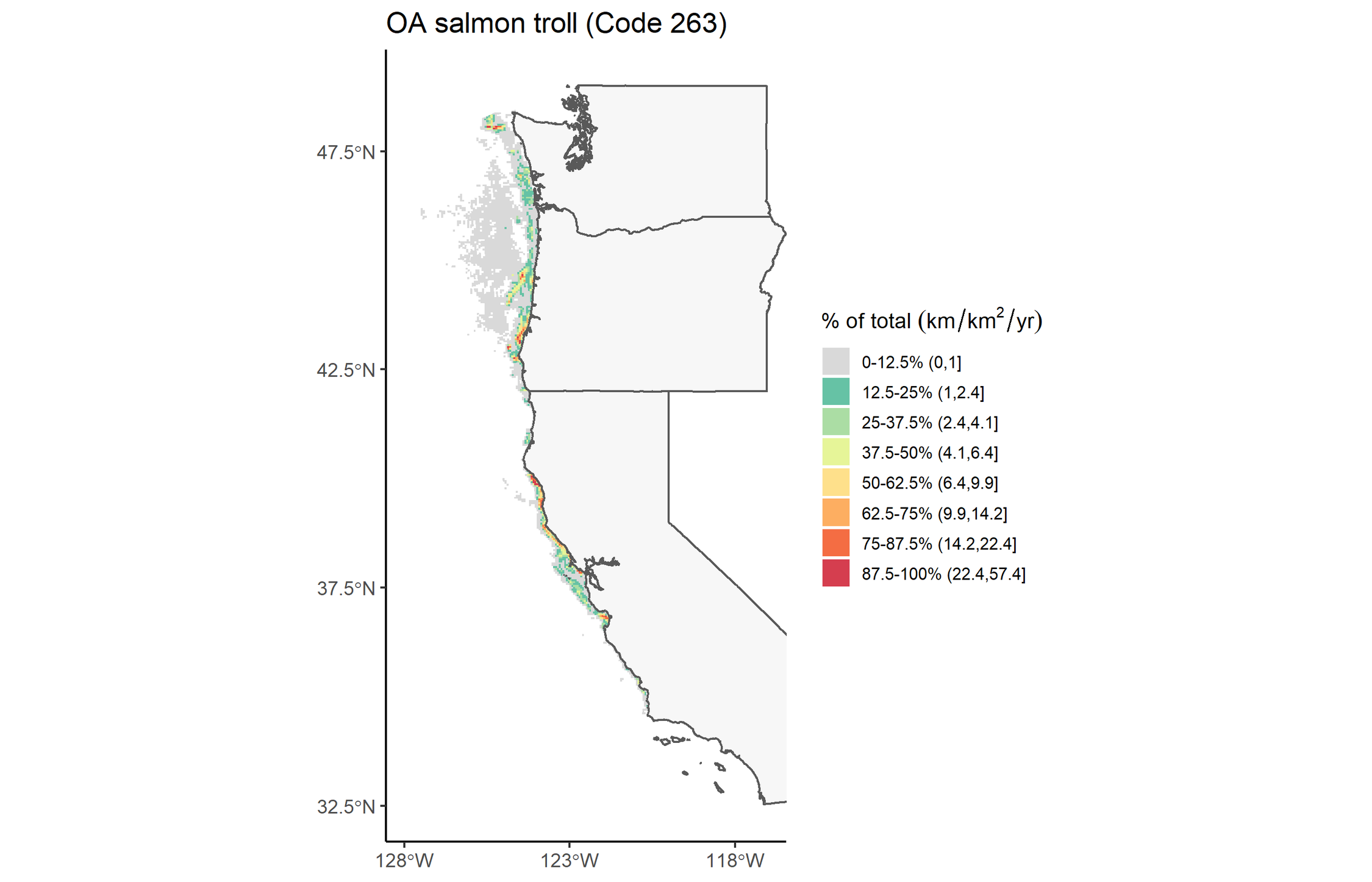

Supplement: S20 Fig — An isobath line is not added since it is not applicable to the salmon troll fishery. (TIF) [file pone.0298868.s020.tif]

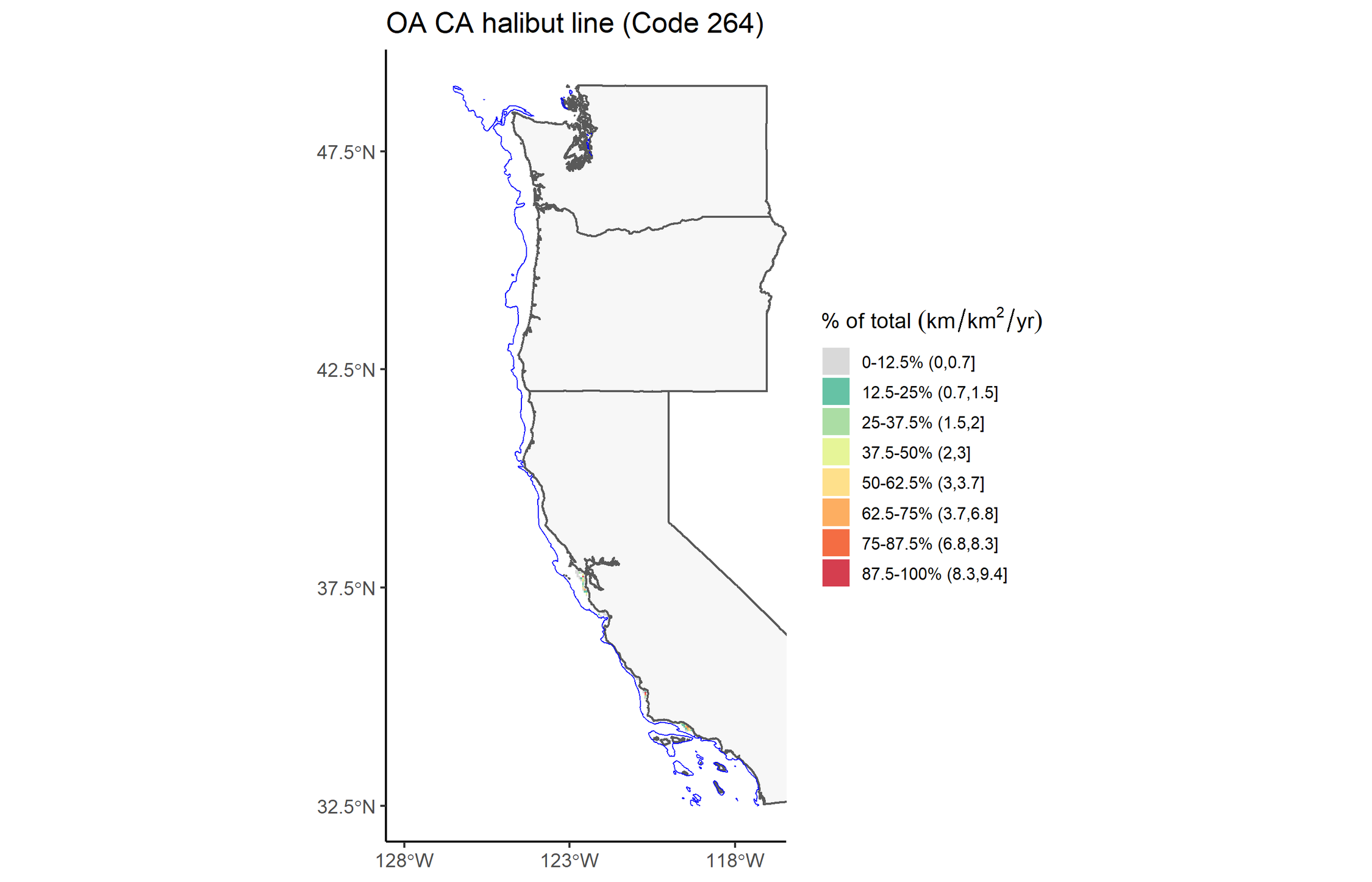

Supplement: S21 Fig — The blue line is the 200 m isobath, representing the maximum biological depth for the target species. The area outside of the biological depth limit may represent erroneous declaration codes, and only represent 6.3% of the total effort for the fishery. (TIF) [file pone.0298868.s021.tif]

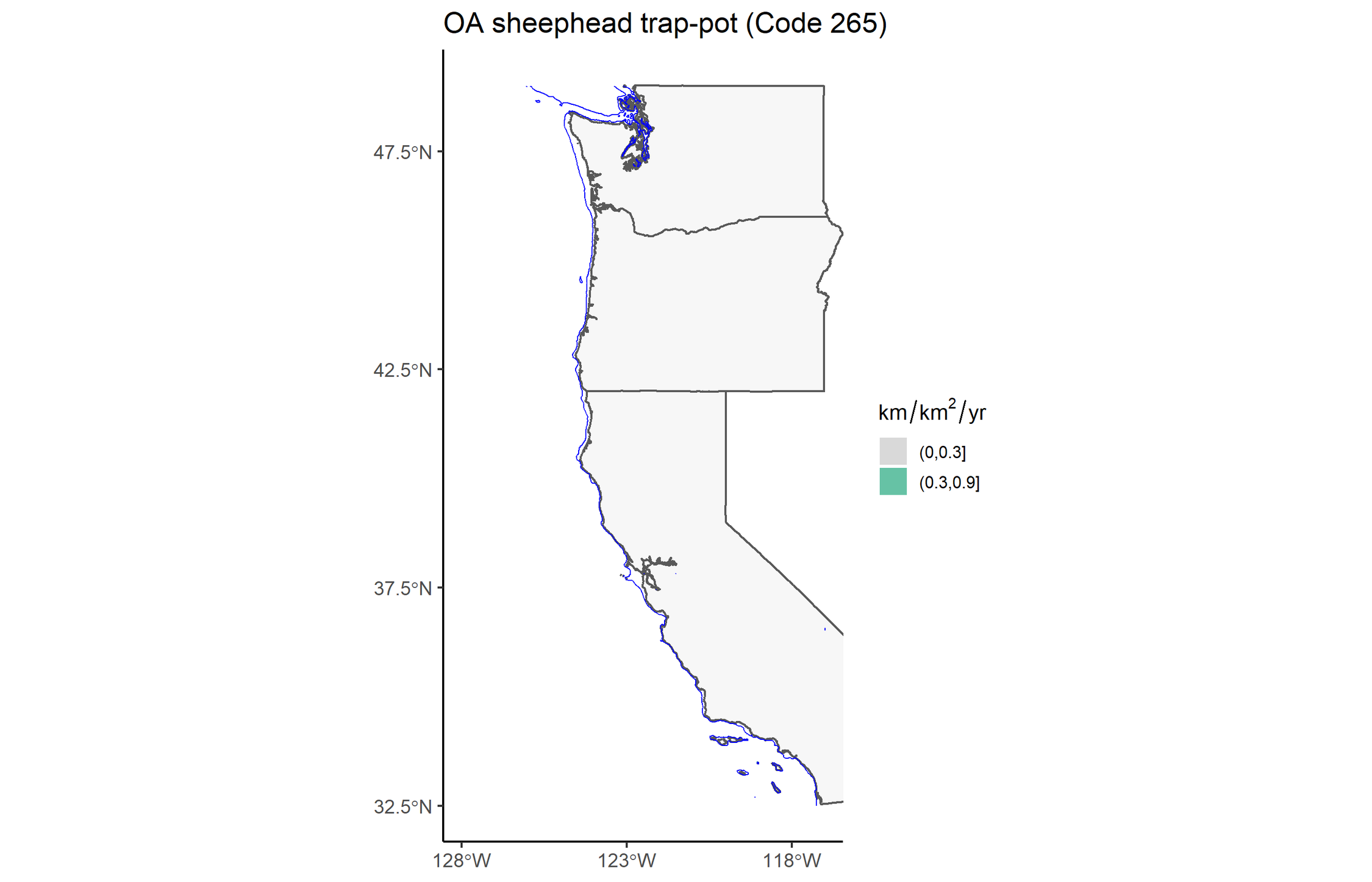

Supplement: S22 Fig — The blue line is the 60 m isobath, representing the maximum biological depth for the target species. The area outside of the biological depth limit may represent erroneous declaration codes, and represent 26.2% of the total effort for the fishery. Data were too sparse to generate octiles as in the other plots. (TIF) [file pone.0298868.s022.tif]

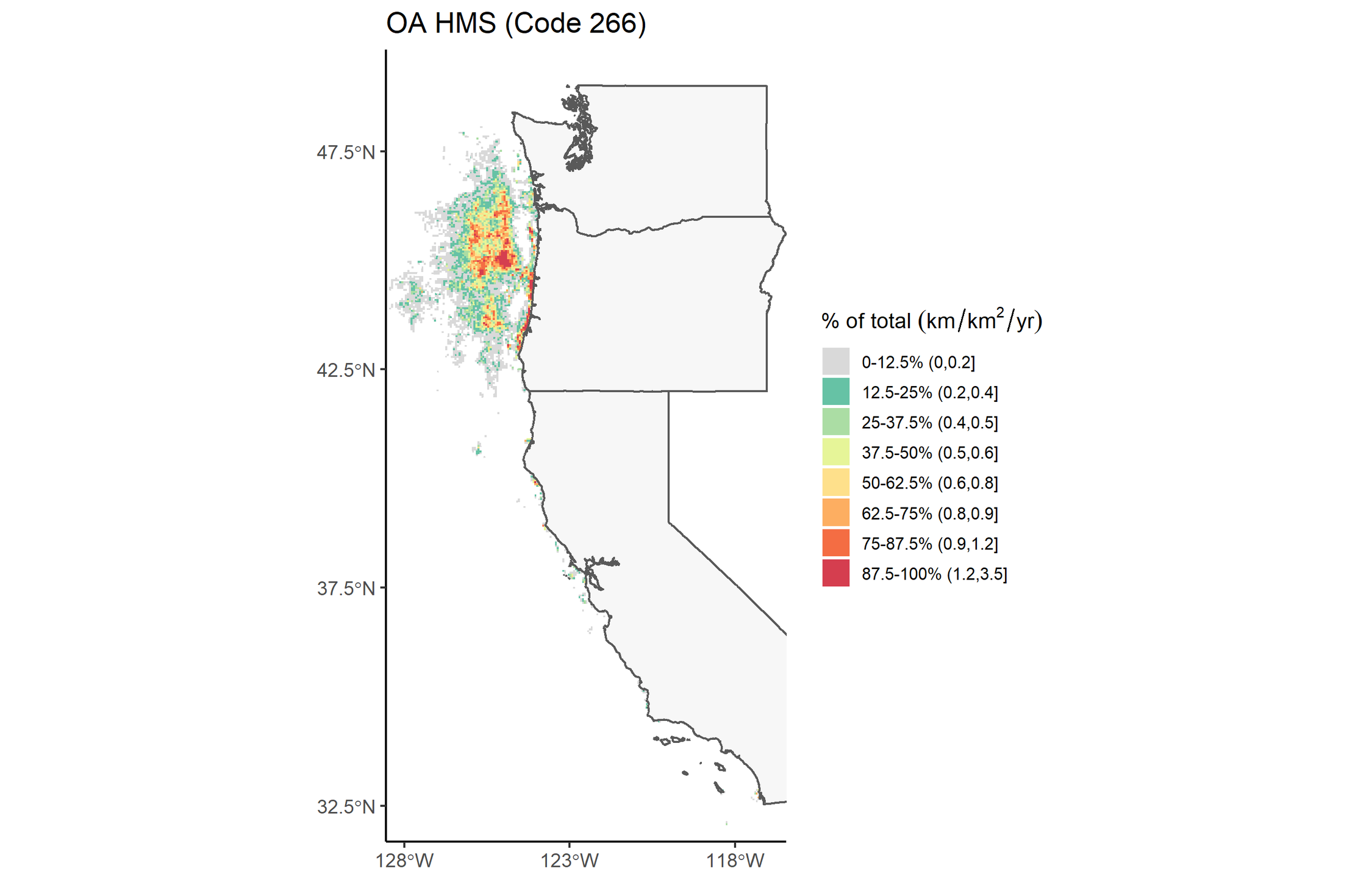

Supplement: S23 Fig — An isobath line is not added since it is not applicable to the HMS fishery. (TIF) [file pone.0298868.s023.tif]

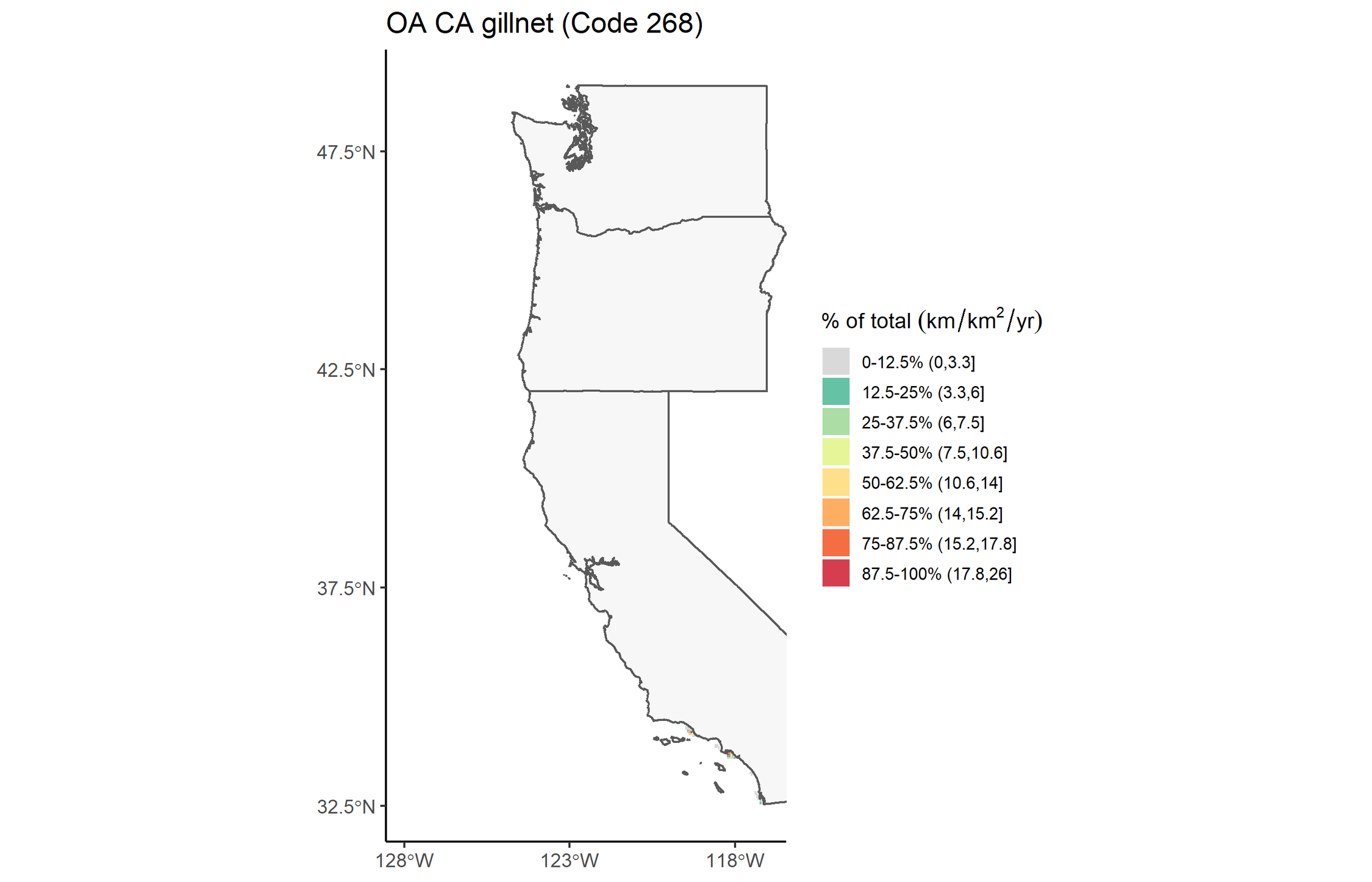

Supplement: S24 Fig — An isobath line is not added since it is not applicable to the gillnet fishery. (TIF) [file pone.0298868.s024.tif]

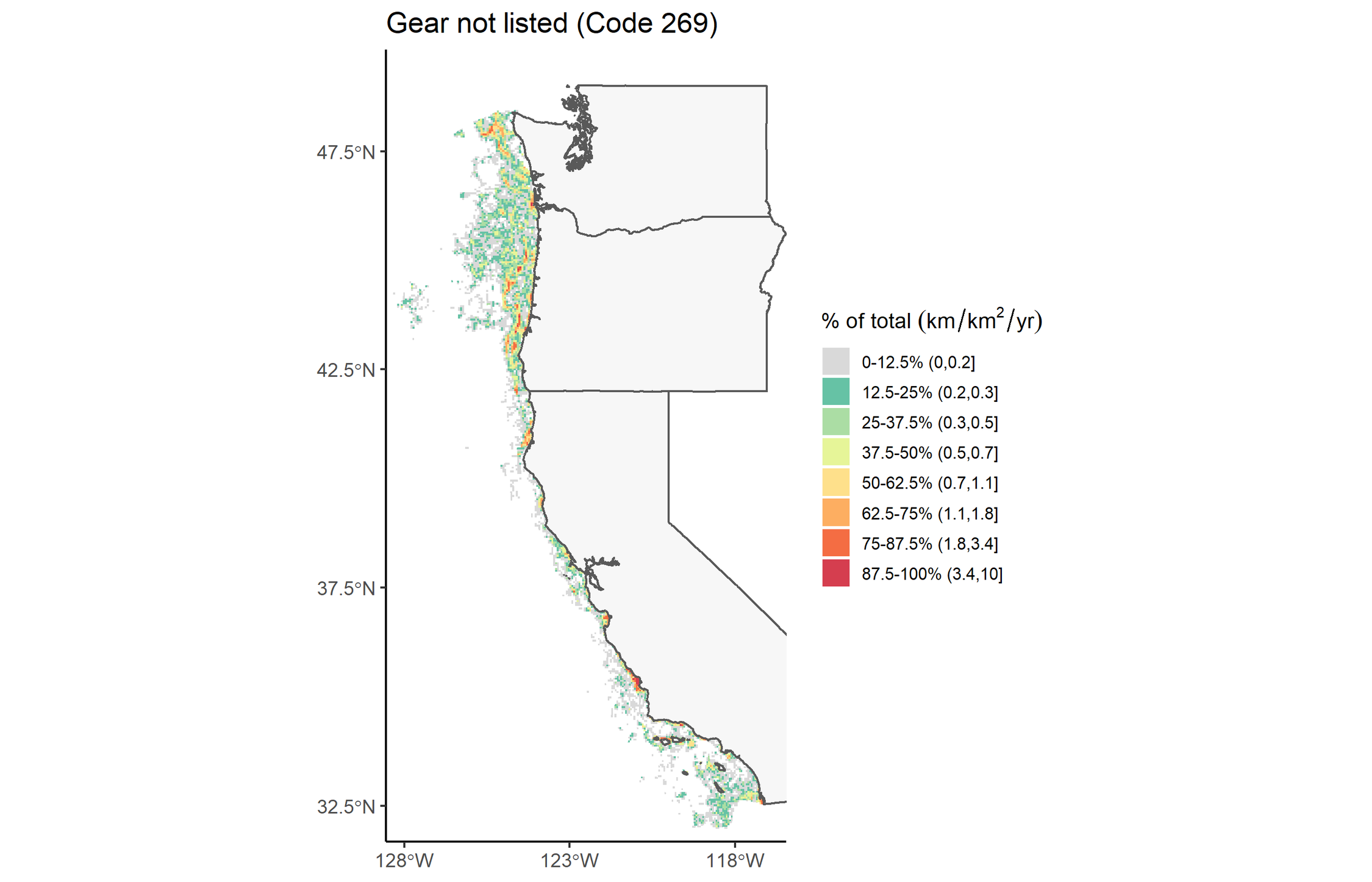

Supplement: S25 Fig — An isobath line is not added since it is unknown what species are captured with unlisted gear. (TIF) [file pone.0298868.s025.tif]

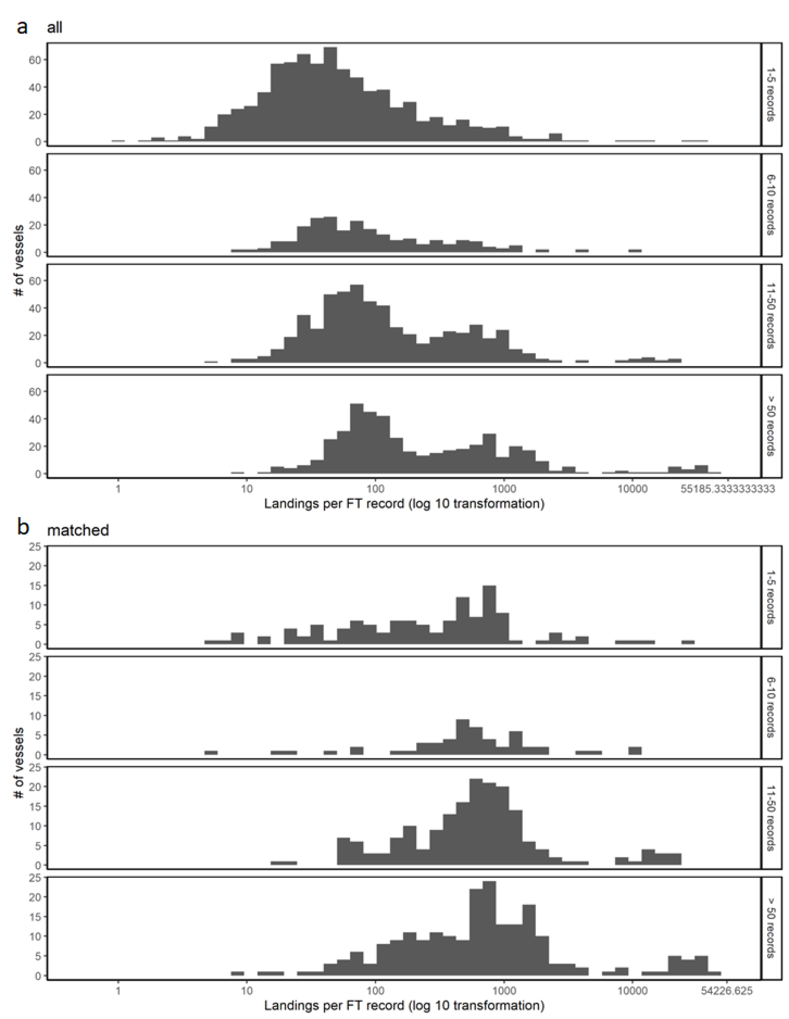

Supplement: S26 Fig — Panels from top to bottom represent vessels reporting fish total ticket records between 1 and 5, between 6 and 10, between 11 and 50, and greater than 50. (a) Total fish ticket records. (b) Fish ticket records that matched with VMS (matched). The unit of landings is pounds. Note that the vessels that reported 5 or fewer fish tickets also reported lower landings per fish ticket than those that reported >50 fish tickets. That is, vessels that reported a relatively small number of fish tickets tended to also catch less fish per trip. (TIF) [file pone.0298868.s026.tif]

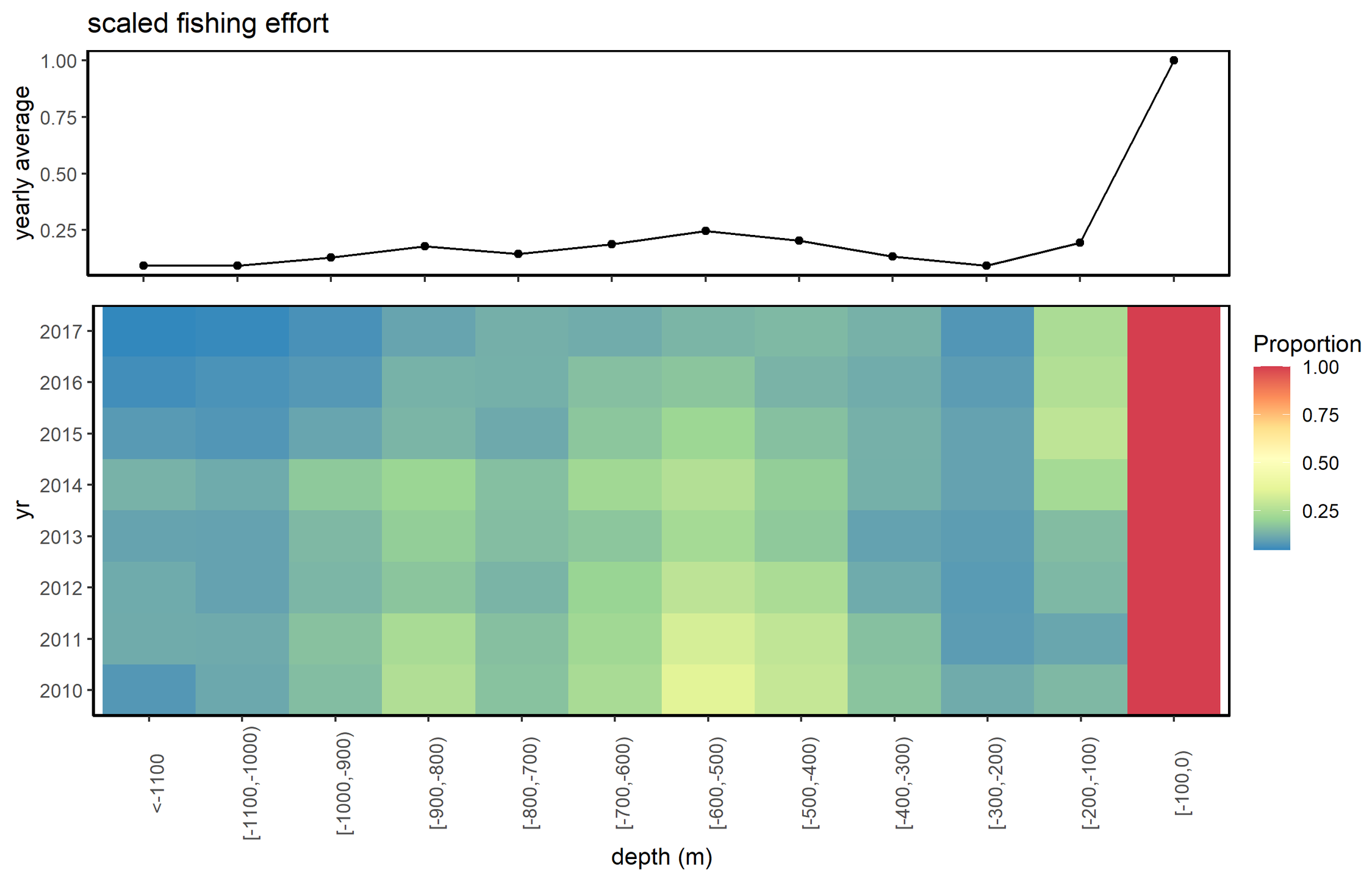

Supplement: S27 Fig — The top panel shows the average of the scaled fishing effort in the bottom panel over years. (TIF) [file pone.0298868.s027.tif]

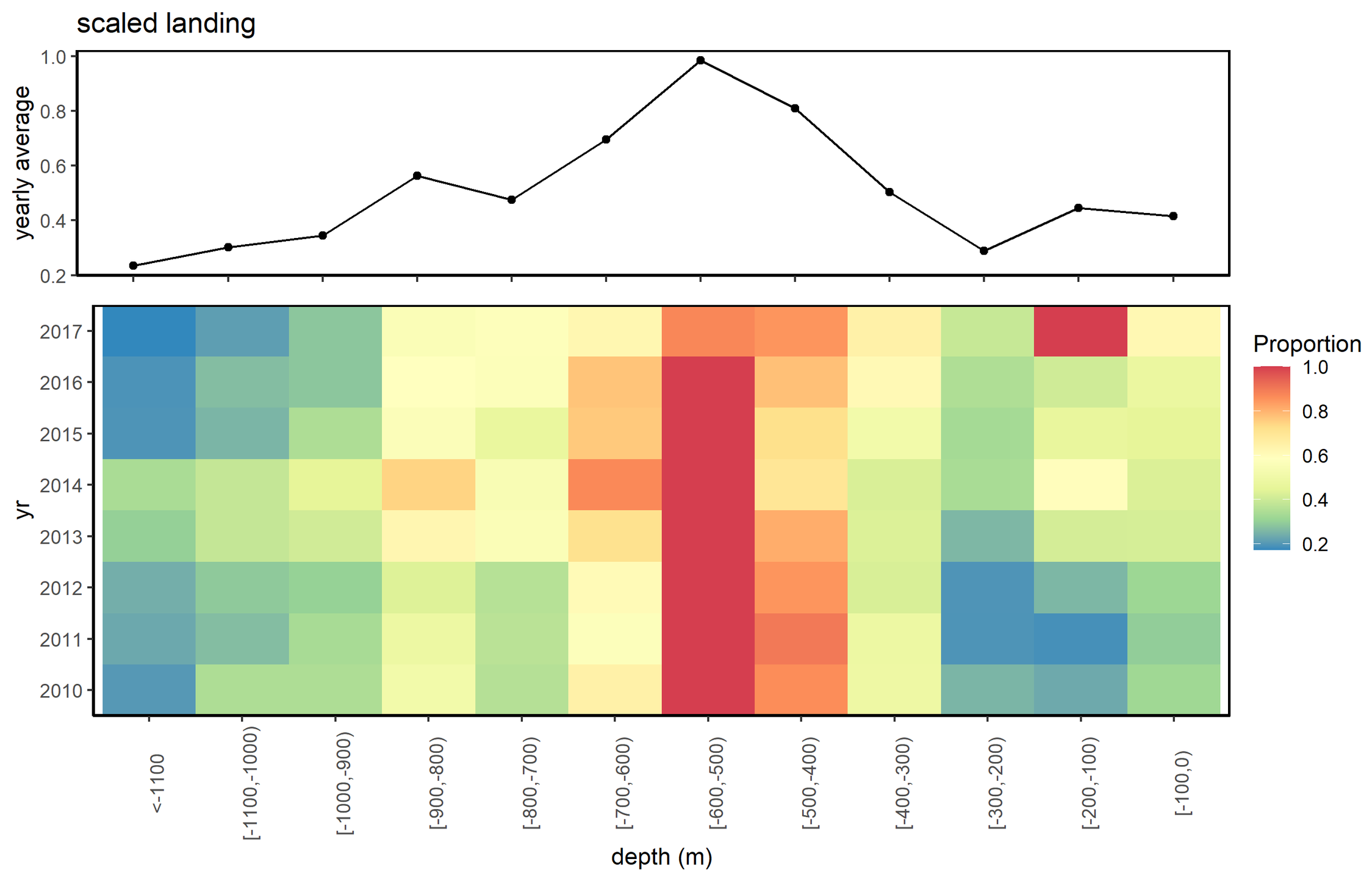

Supplement: S28 Fig — (TIF) [file pone.0298868.s028.tif]

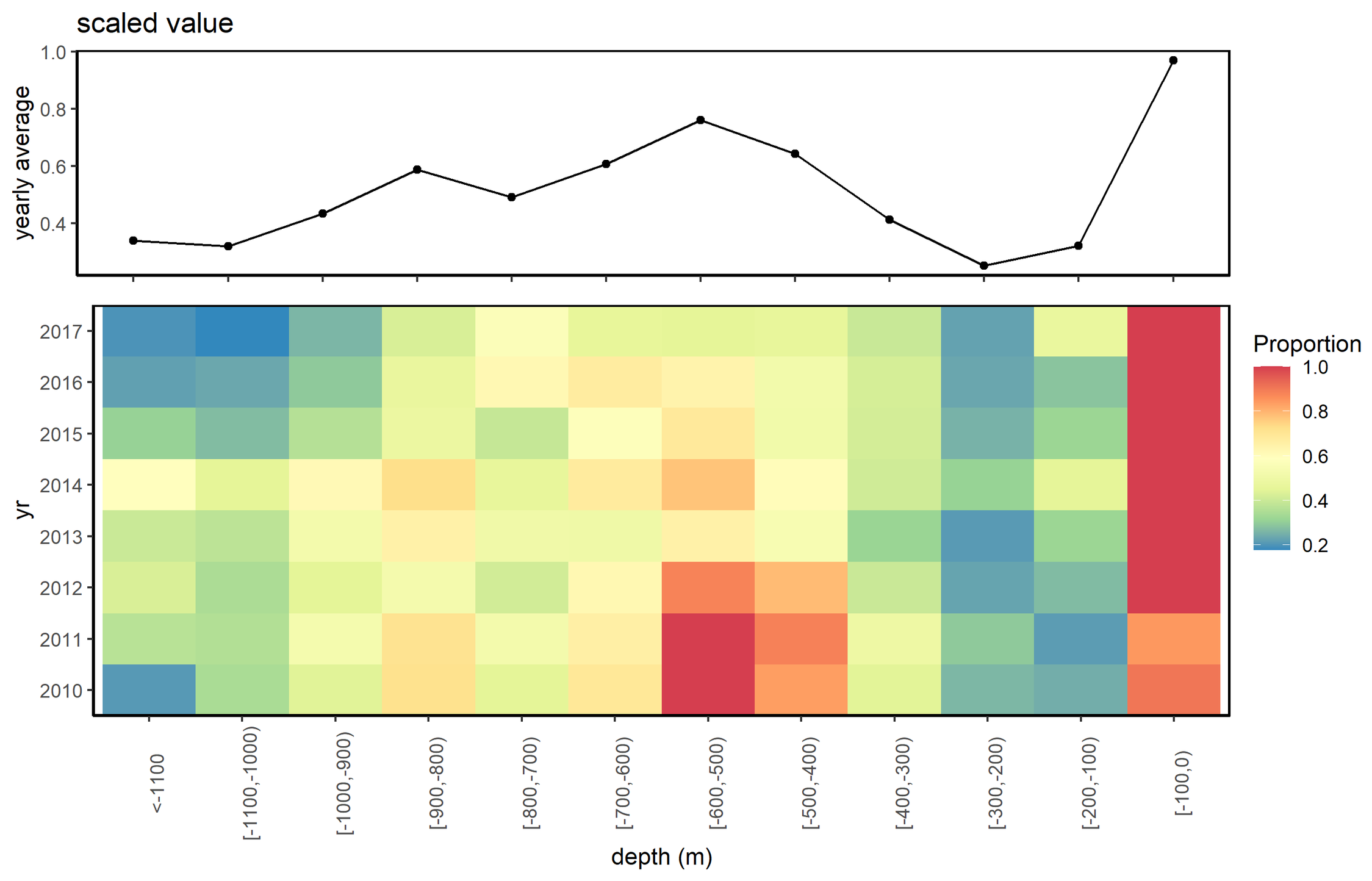

Supplement: S29 Fig — (TIF) [file pone.0298868.s029.tif]

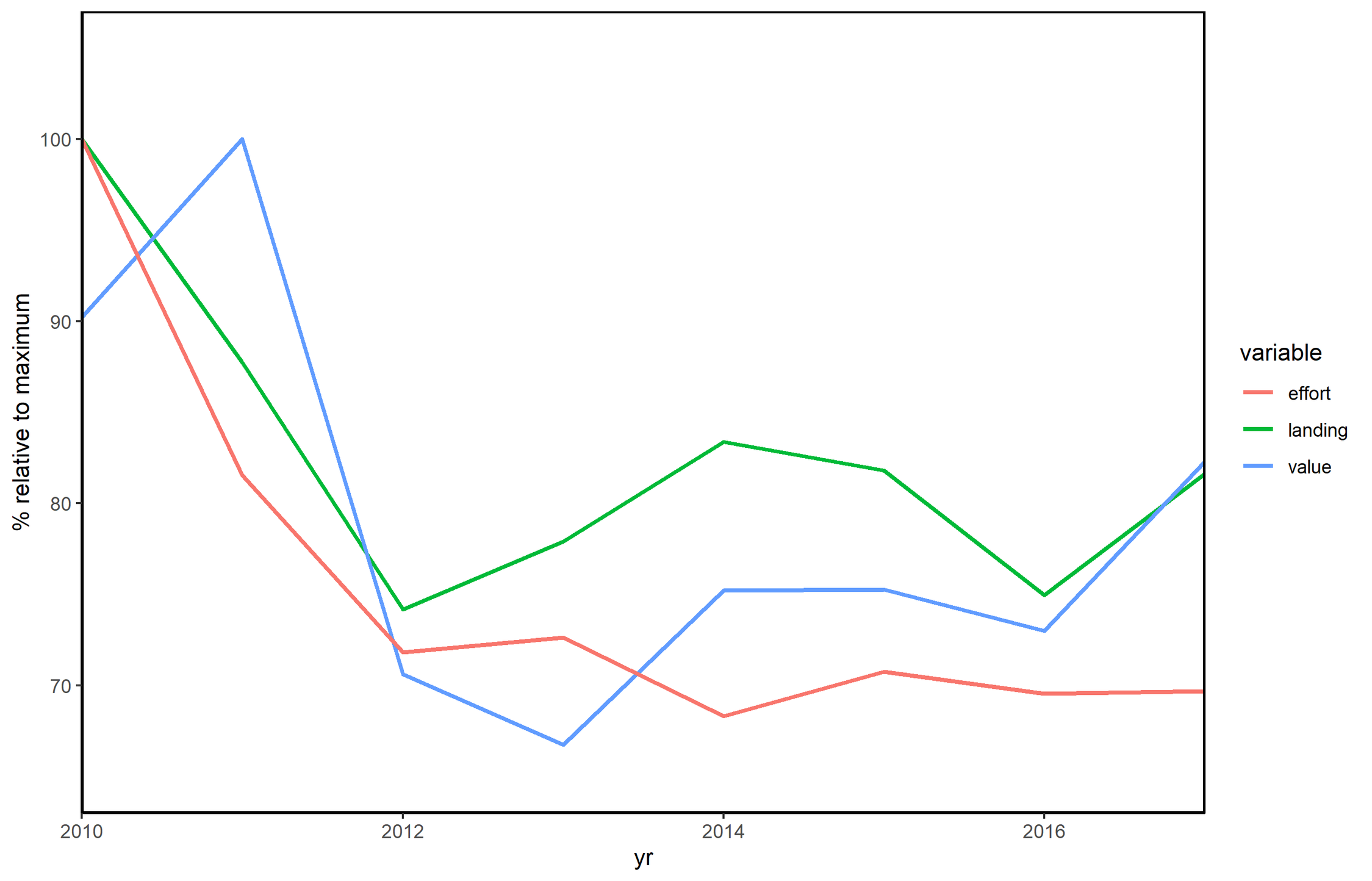

Supplement: S30 Fig — The maximum for effort (red) is 187.89 million km for effort in 2010. Based on VMS-FT data for groundfish, the maximum for landings (green) is 14.66 million pounds for landing in 2010. The maximum for ex-vessel value (blue) is $23.23 million in 2011. (TIF) [file pone.0298868.s030.tif]
